# A Quasi-Experimental Study of Medicaid Expansion and Urban Mortality in the American Northeast

**DOI:** 10.3389/fpubh.2021.707907

**Published:** 2021-11-17

**Authors:** Cyrus Ayubcha, Pedram Pouladvand, Soussan Ayubcha

**Affiliations:** ^1^Harvard Medical School, Boston, MA, United States; ^2^Alfred I. DuPont Hospital for Children, Wilmington, NC, United States; ^3^Marcus Institute of Integrative Health, Thomas Jefferson University, Philadelphia, PA, United States

**Keywords:** Medicaid expansion, Medicaid, cities, mortality, urban

## Abstract

**Objectives:** To investigate the association of state-level Medicaid expansion and non-elderly mortality rates from 1999 to 2018 in Northeastern urban settings.

**Methods:** This quasi-experimental study utilized a synthetic control method to assess the association of Medicaid expansion on non-elderly urban mortality rates [1999–2018]. Counties encompassing the largest cities in the Northeastern Megalopolis (Washington D.C., Baltimore, Philadelphia, New York City, and Boston) were selected as treatment units (*n* = 5 cities, 3,543,302 individuals in 2018). Cities in states without Medicaid expansion were utilized as control units (*n* = 17 cities, 12,713,768 individuals in 2018).

**Results:** Across all cities, there was a significant reduction in the neoplasm (Population-Adjusted Average Treatment Effect = −1.37 [95% CI −2.73, −0.42]) and all-cause (Population-Adjusted Average Treatment Effect = −2.57 [95%CI −8.46, −0.58]) mortality rate. Washington D.C. encountered the largest reductions in mortality (Average Treatment Effect on All-Cause Medical Mortality = −5.40 monthly deaths per 100,000 individuals [95% CI −12.50, −3.34], −18.84% [95% CI −43.64%, −11.67%] reduction, *p* = < 0.001; Average Treatment Effect on Neoplasm Mortality = −1.95 monthly deaths per 100,000 individuals [95% CI −3.04, −0.98], −21.88% [95% CI −34.10%, −10.99%] reduction, *p* = 0.002). Reductions in all-cause medical mortality and neoplasm mortality rates were similarly observed in other cities.

**Conclusion:** Significant reductions in urban mortality rates were associated with Medicaid expansion. Our study suggests that Medicaid expansion saved lives in the observed urban settings.

## Introduction

The Affordable Care Act (ACA) offered states the opportunity to expand health insurance coverage to non-elderly adult populations through Medicaid expansion (ME). States were able to use federal funding to increase state Medicaid coverage to all those US Citizens and permanent residents with incomes at or below 138% federal poverty level (FPL) (1). Specific narrow categories of eligibility (e.g., impoverished pregnant women) were federally mandated earlier. Prior to the 2014 implementation of ACA Medicaid expansion, some states utilized waivers to preemptively expand their programs sometimes with more expansive eligibility criteria, but significant gaps in coverage persisted (2). As a result of Medicaid expansion, Medicaid take-up increased in less-educated, low-income, minority, and younger adults residing in expansion states as compared to peers in nonexpanded states (3). What remains unclear is whether this increase in coverage improved health outcomes, particularly whether urban settings observed reductions in mortality.

Cost-benefit considerations, entailing monetary cost, value of increasing coverage, and the quality of care provided to beneficiaries, have been of notable interest in the debate to increase public medical coverage (4). The original Medicaid program has been associated with moderate decreases in mortality depending on the methods employed (5). Expansions of Medicaid to pregnant women and children during the 1980s were linked to decreases in infant mortality and maternal mortality in most studies (6, 7). Analyses of the 2006 Massachusetts Health Care Reform (MHCR) found reduced all-cause mortality by nearly 8.2 deaths per 100,000 adults (8–10). However, the Oregon Healthcare Experiment study suggested that the effects of coverage may not be immediate or large (11). Such variability may suggest the mechanisms and impact of health coverage is contingent upon alternative factors (e.g., urban-rural residence, minority identity, socioeconomic status, etc.); specific benefits to certain sub-populations or temporally removed effects may further underlie such variability. Smaller mortality effects may also be challenging to capture as gains in public insurance can often be centered in younger populations though older populations are most likely to benefit from coverage.

A number of studies have examined the ACA Medicaid expansion directly and these studies also gesture toward effect heterogeneity in increasing coverage. Nationwide studies observed a decline in all-cause mortality following the ACA Medicaid expansions, but not in cause-specific mortality rates (cardiovascular, respiratory, suicide, and opioid overdose) (12) while a separate analysis found only small, insignificant effects (13). Emerging evidence suggests that Medicaid expansion may have reduced excessive mortality for minorities, in part, by reducing amenable mortality (10, 14). These reforms also seem to have reduced maternal mortality rates, particularly for late-maternal deaths and Black mothers (15, 16). Some studies have observed improvements in specific mortality measures, such as cardiovascular mortality in near-elderly populations and one-year mortality rates among end-stage renal disease patients (17, 18).

We examine the influence of Medicaid expansion on the mortality rates in various Northeastern urban centers. Urban populations warrant targeted study as they are largely distinct from state-wide populations with respect to diversity, healthcare access barriers, types of disease burden and disparities; this is not to mention that a majority of Americans reside in urban settings (19–24). Notably, most large American metropolitan centers are unique in characteristics and state policy history which presents difficulties in studying the generalized “urban” populations. This motivates our study to take a narrow and detailed examination into each city to determine the impact of Medicaid expansion. Accordingly, the observed changes across the included cities may be a means to logically deducing certain city-specific environmental factors that underlie treatment effects. Our study does not only examine all-cause medical mortality but also certain cause-specific mortality rates (e.g., circulatory mortality, neoplasm mortality, etc.). This study will help to understand whether expanding medical coverage can reduce all-cause and cause-specific urban mortality rates, thus helping illuminate why health coverage may improve health for some but not all.

## Materials and Methods

### Study Design and Inclusion Criteria

We utilized a quasi-experimental design to assess the mortality rate among those aged 20–64 from 1999 to 2018; where observed mortality rates after Medicaid expansion were compared to respective predicted mortality rates for each treated city. Only cities in the Northeast megalopolis were considered when selecting treated urban counties. The first year of treatment for each treated city unit was considered the year in which statewide Medicaid expansion was enacted. Urban counties within non-expansion states were selected for the control group. All urban counties with sufficient population levels (>9,000 individuals) and population density and (>800 individuals/mi2) were included. States and counties with any previous Federal Poverty Level (FPL)-based waiver expansions were eliminated from this control pool (e.g., Wisconsin, St. Louis City, MO). The counties utilized in this study can be found in Table 1.

**Table 1:** Treatment conditions of included cities.

**City**	**County**	**State**	**Expansion year**	**Expansion FPL**	**Treatment**
Baltimore^†^	Baltimore City	MD	2014	138%	Treatment
San Antonio	Bexar	TX	None	None	Control
Fort Lauderdale	Broward	FL	None	None	Control
Dallas	Dallas	TX	None	None	Control
Nashville	Davidson	TN	None	None	Control
Washington D.C.^††^	District of Columbia		2010	210%	Treatment
Atlanta	Fulton	GA	None	None	Control
Houston	Harris	TX	None	None	Control
Kansas City	Johnson	KS	None	None	Control
Charlotte	Mecklenburg	NC	None	None	Control
Memphis	Shelby	TN	None	None	Control
Miami	Miami-Dade	FL	None	None	Control
New York City^‡^	New York	NY	2014	138%	Treatment
Oklahoma City	Oklahoma	OK	None	None	Control
Orlando	Orange	FL	None	None	Control
Philadelphia	Philadelphia	PA	2015	138%	Treatment
Tampa St. Petersburg Clearwater	Pinellas	FL	None	None	Control
Salt Lake City	Salt Lake	UT	None	None	Control
Boston^‡‡^	Suffolk	MA	2014	138%	Treatment
Fort Worth	Tarrant	TX	None	None	Control
Austin	Travis	TX	None	None	Control
Raleigh	Wake	NC	None	None	Control

† *07/01/06 § 1115 Waiver that established the Primary Adult Care program to expand coverage (prescription, primary care, behavioral health) to childless adults at or below 116% of FPL*.

†† *07/01/10 State Plan Amendment extends Medicaid coverage to 133% FPL | 12/01/10 § 1115 Waiver Early ACA expansion extends Medicaid program to 210% of FPL*.

‡ *10/01/01 § 1115 Waiver extends Medicaid Family Health Plus to childless adults at 100% FPL*.

‡‡ *4/6/06 Massachusetts implemented reforms to expand insurance coverage to low- income adults beginning in 2006*.

### Data Sources

All data were secondary, public, and de-identified; no institutional review board approval or informed consent was required. County-level age-adjusted mortality data for individuals between the ages of 20–64 were compiled from the Centers for Disease Control WONDER Tool. The following categories of mortality were included: diseases of the circulatory system mortality (circulatory mortality), diseases of the respiratory system mortality (respiratory mortality), all-cause medical mortality, and neoplasm mortality (i.e., cancer, malignancy). All-cause medical mortality was defined as all-cause mortality absent external-cause mortality. Categories of mortality were defined by International Classification of Diseases (ICD) coding systems; the ICD 9 to ICD 10 code transition was reconciled.

Several longitudinal county-level covariates were obtained and utilized as the basis for developing the synthetic control for each treatment city. From 1999 to 2018, healthcare coverage rates were attained from the Small Area Health Insurance Estimates, non-Hispanic white percentages of populations between 20 and 64 were calculated using the Bridged-Race Population Estimates, inflation-adjusted median income and poverty rates were captured in data from the Bureau of Economic Analysis and the Small Area Income and Poverty Estimates. The Economic Research Services of the United States Department of Agriculture provided educational attainment fractions as defined as the fraction of those with at least some college. Unavailable covariate data for health insurance coverage (1999–2005) were interpolated using only the trends provided by five-year American Community Survey estimates and Census data.

### Main Data Outcomes

Mortality was acquired from 1999 to 2018 and all rates were calculated as age-adjusted deaths per 100,000 population of 20–64-year-olds in the county. Monthly data were used for all-cause medical mortality and neoplasm mortality rates, while yearly data were used for all other forms of mortality; more granular data (i.e., monthly as opposed to yearly) provided greater statistical power but less common forms of mortality lacked sufficient prevalence to justify monthly analyses in light of limited sample sizes and elevated risk of stochasticity.

### Statistical Analysis

This study employs synthetic control methods which are advantageous when no single control unit can serve as an ideal comparator for a treated unit. This application creates a synthetic control city unit through a weighted combination of the control cities. The synthetic city is intended to simulate the mortality rate of the treated city (e.g., Philadelphia) in the post-treatment period (e.g., 2015–2018) if Medicaid had not been expanded. The particular weights varied in the specific model as applied to each treated city; selection of weights chiefly aims to create a synthetic city with similar pre-intervention covariate characteristics (e.g., inflation-adjusted median income, lagged mortality rates) to the treated city; this algorithmic process employed by the GSC is akin to creating a control city with similar characteristics to the treated city. The appropriateness of the synthetic city as a comparator to the treated city is determined by the convergence of the pre-intervention outcome trends between the treated city and the synthetic city. Such that, any divergence of trends in the post-intervention can be attributed to the intervention (i.e., Medicaid expansion).

In this study, we utilize a Generalized Synthetic Control (GSC) model (25) for each treated city and specific mortality rate. The GSC model relies upon an interactive fixed effects (IFE) technique within the synthetic control framework. The use of IFE incorporates two-way fixed effects which enabled our model to account for unobserved unit-specific and time-specific confounding variables. IFE modeling of the control units is then used to create out-of-sample predictions for the treated unit which results in a GSC output of the synthetic city's mortality rates. The difference between the synthetic and treatment mortality outcomes is considered the treatment effect on the treated unit. The post-intervention difference between the trends can be averaged over the post-treatment period; termed the average treatment effected in the treated units (ATT). Parametric bootstrapping inference tests were applied to ascertain uncertainty estimates (25).

One sensitivity test was performed by rerunning the models of the monthly data in yearly format to determine whether the structure of yearly analyses influenced the model. An in-time placebo sensitivity analysis was performed applying a false intervention point in the middle of the pre-intervention period and running the GSC model through the pre-intervention period; specifically, the pre-treatment period was considered 1999–2005 and the post-treatment period was considered 2006–2007 so that the 2008 economic downturn was not included. Given that insurance rate is considered a significant mediating variable, a secondary analysis was also performed utilizing county-level insurance rates for each of the treatment cities. The GSC model was applied to insurance coverage rates as a secondary outcome using all the predictors utilized in the main models except for mortality data. All statistical analyses were conducted in R (version 4.0.0) using the gsynth package.

The GSC overcomes some limitations of alternative methods. The GSC does not rely upon the parallel trend assumption that is required in the difference-in-differences methods (25). The GSC method has further proven to be less sensitive to idiosyncratic volatility with a small number of observations (25). Furthermore, this method does not require sensitivity tests of model specifications as a cross-validation procedure selects the optimal number of factors in the IFE model (25). Nevertheless, the GSC model does assume that a stable combination of control units based on the pre-intervention period characteristics of the treatment and control pool can approximate the outcomes of the treated unit in the post-intervention period absent the intervention. Such advantages may achieve similar or superior performance as compared to other methods; several simulated and applied health policy studies comparing IFE, GSC, DiD, and synthetic control methods have found that GSC models perform best (25, 26). Finally, the GSC structure allows for robust parametric bootstrapping inference whereas such quantitative inference is unavailable for traditional synthetic control methods.

## Results

**Figure 1:**
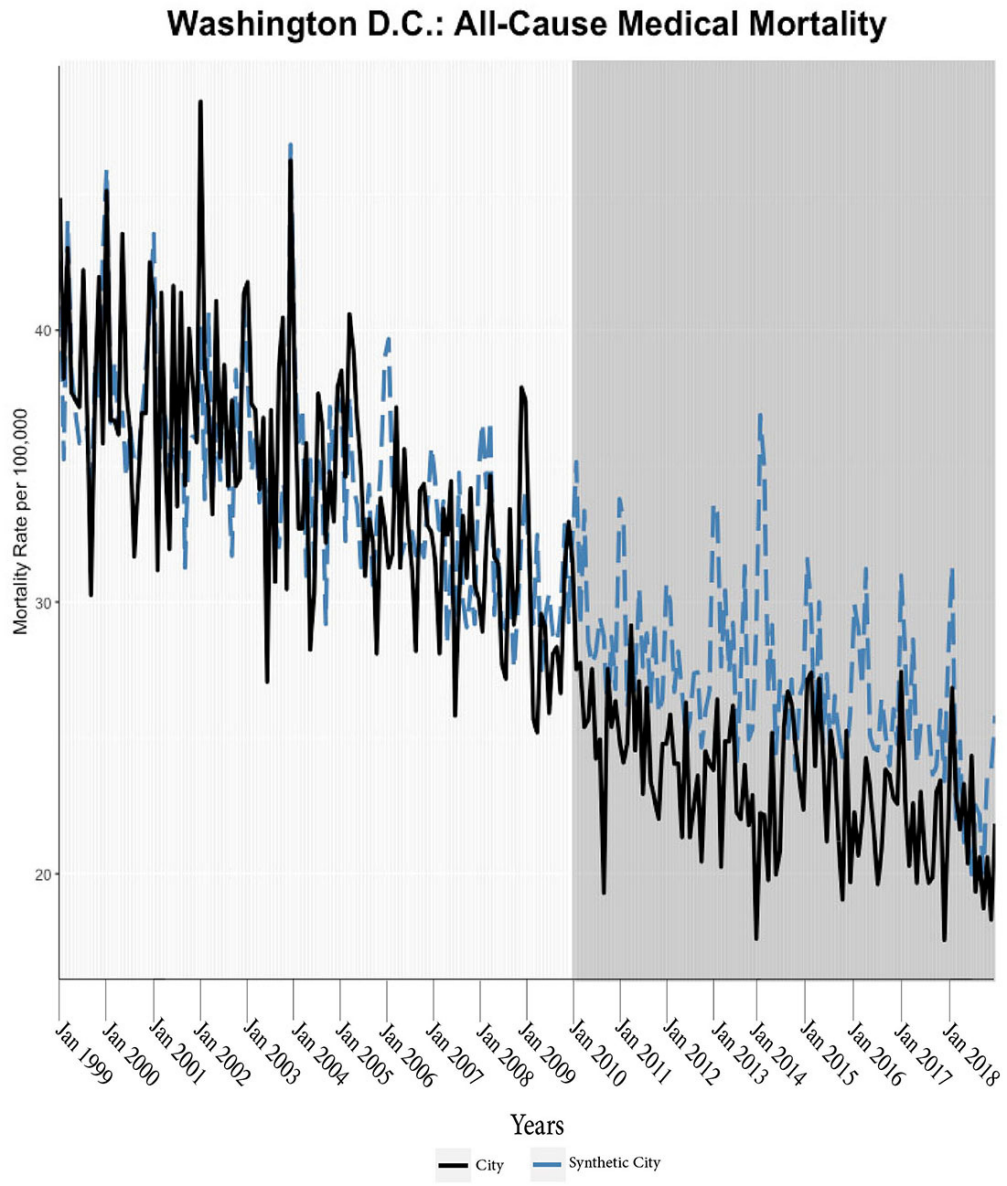
All-cause mortality trends: Washington D.C. and synthetic control city. The blue dotted lines represent the generalized synthetic control prediction (synthetic city) of mortality rates while the solid black line is the observed mortality rate of the Medicaid expansion city. The vertical axis of the graphs represents the per 100,000-person mortality rates. The horizontal axis of the graphs represents time units (i.e., the first month of the year). The darker gray graph areas correlate with the start and duration of Medicaid expansion in the respective city (applicable for all the figures).

**Figure 2:**
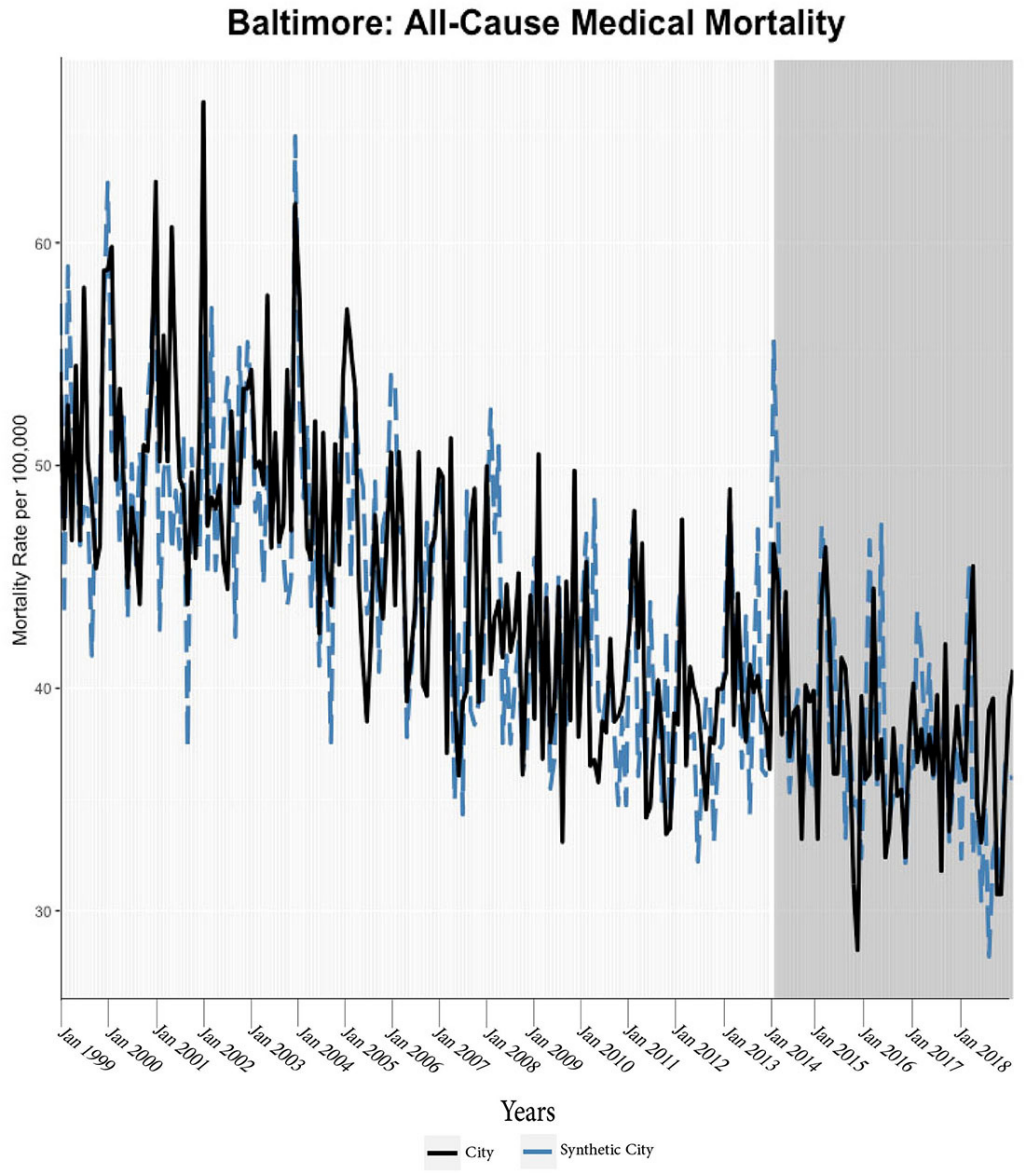
All-cause mortality trends: Baltimore and synthetic control city.

**Figure 3:**
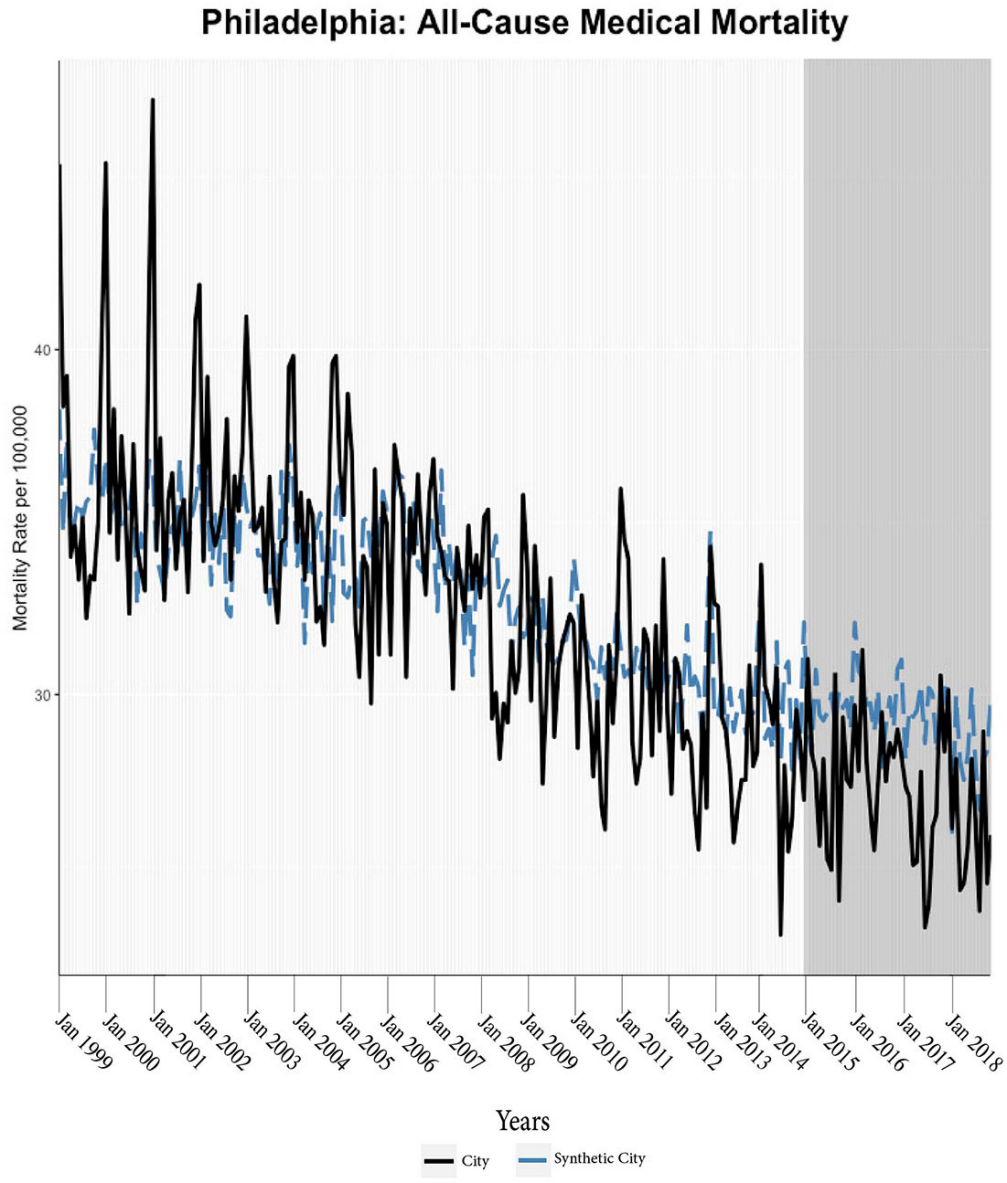
All-cause mortality trends: Philadelphia and synthetic control city.

**Figure 4:**
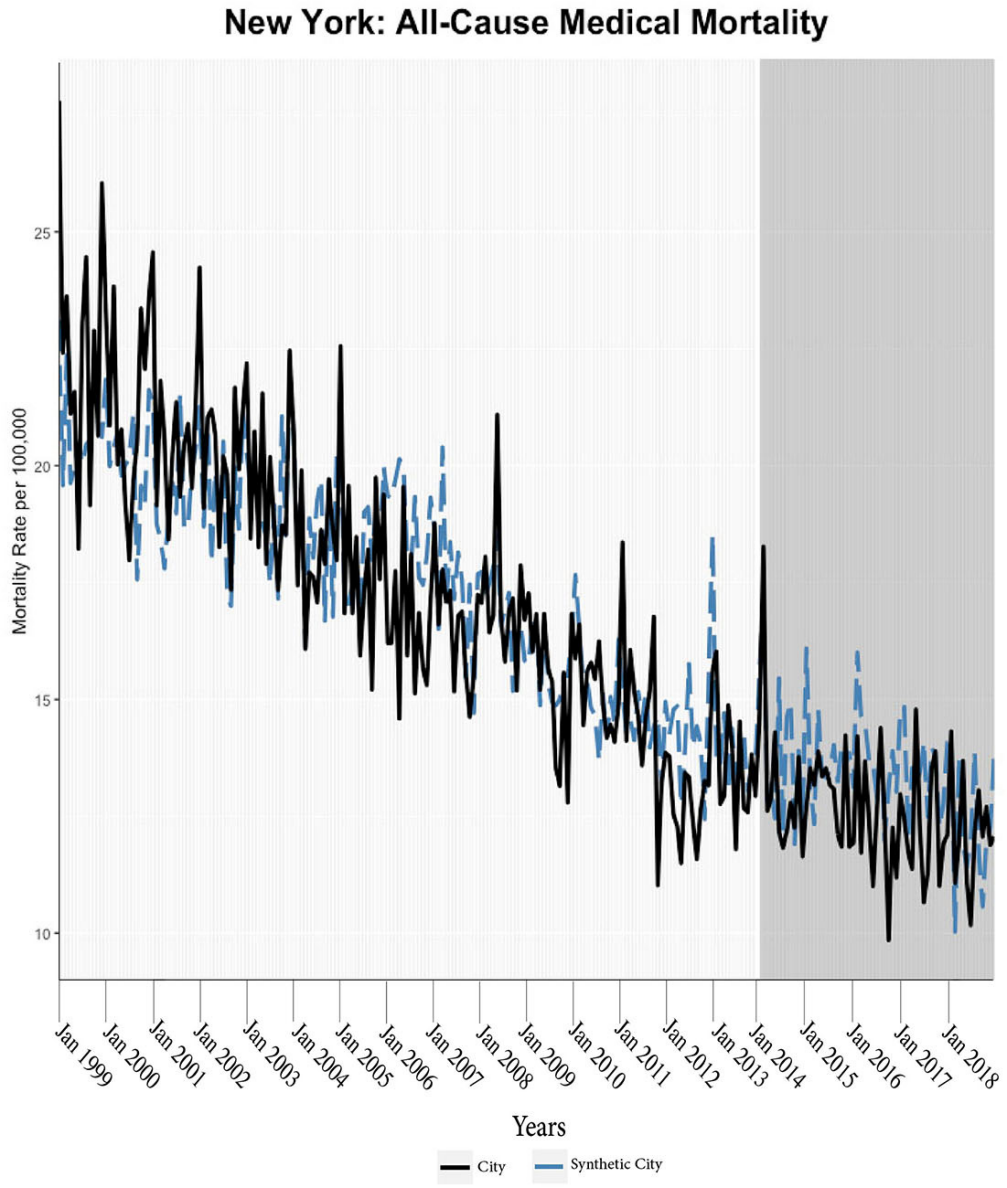
All-cause mortality trends: New York and synthetic control city.

**Figure 5:**
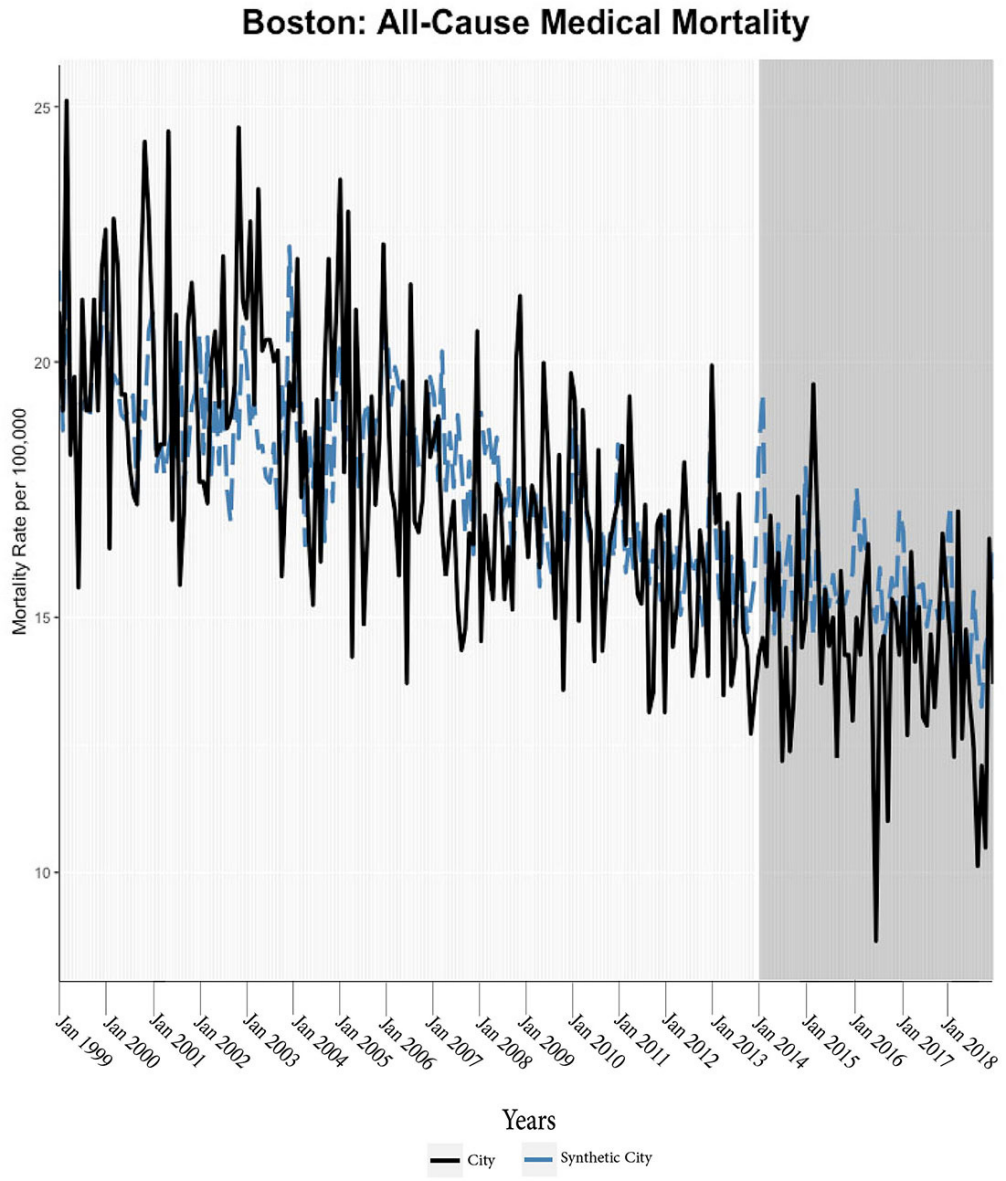
All-cause mortality trends: Boston and synthetic control city.

**Figure 6:**
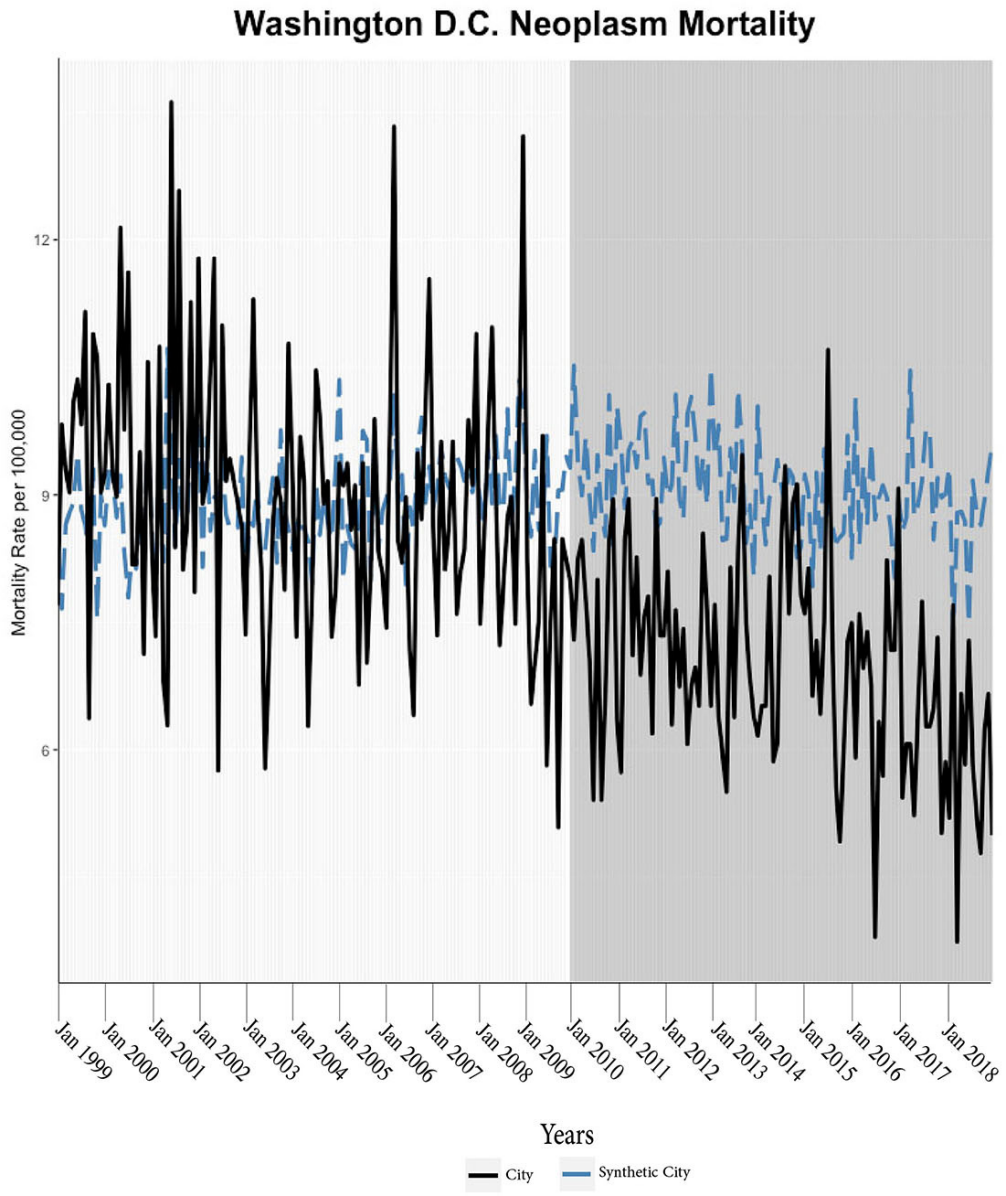
Neoplasm mortality trends: Washington D.C. and synthetic control city.

**Figure 7:**
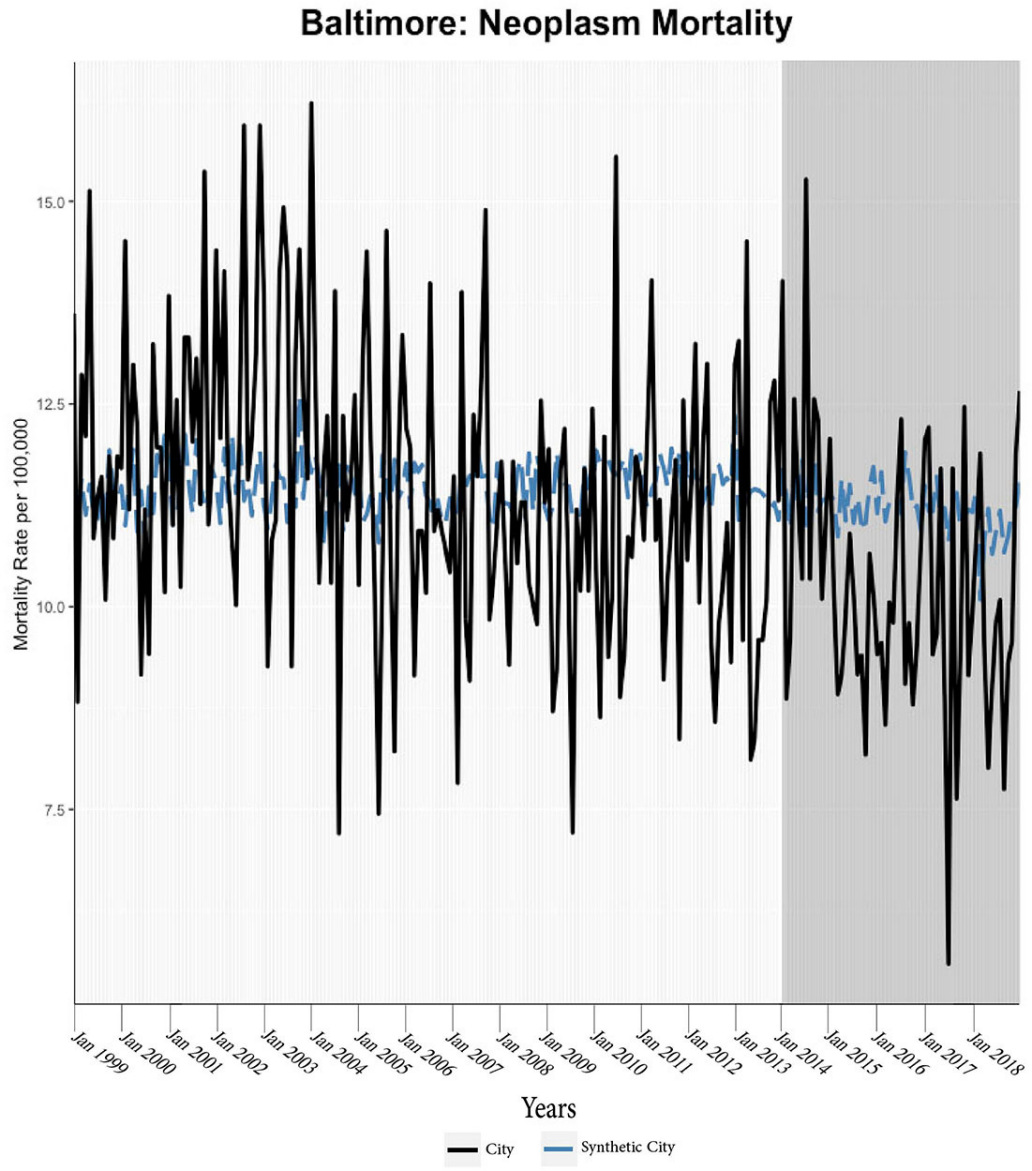
Neoplasm mortality trends: Baltimore and synthetic control city.

**Figure 8:**
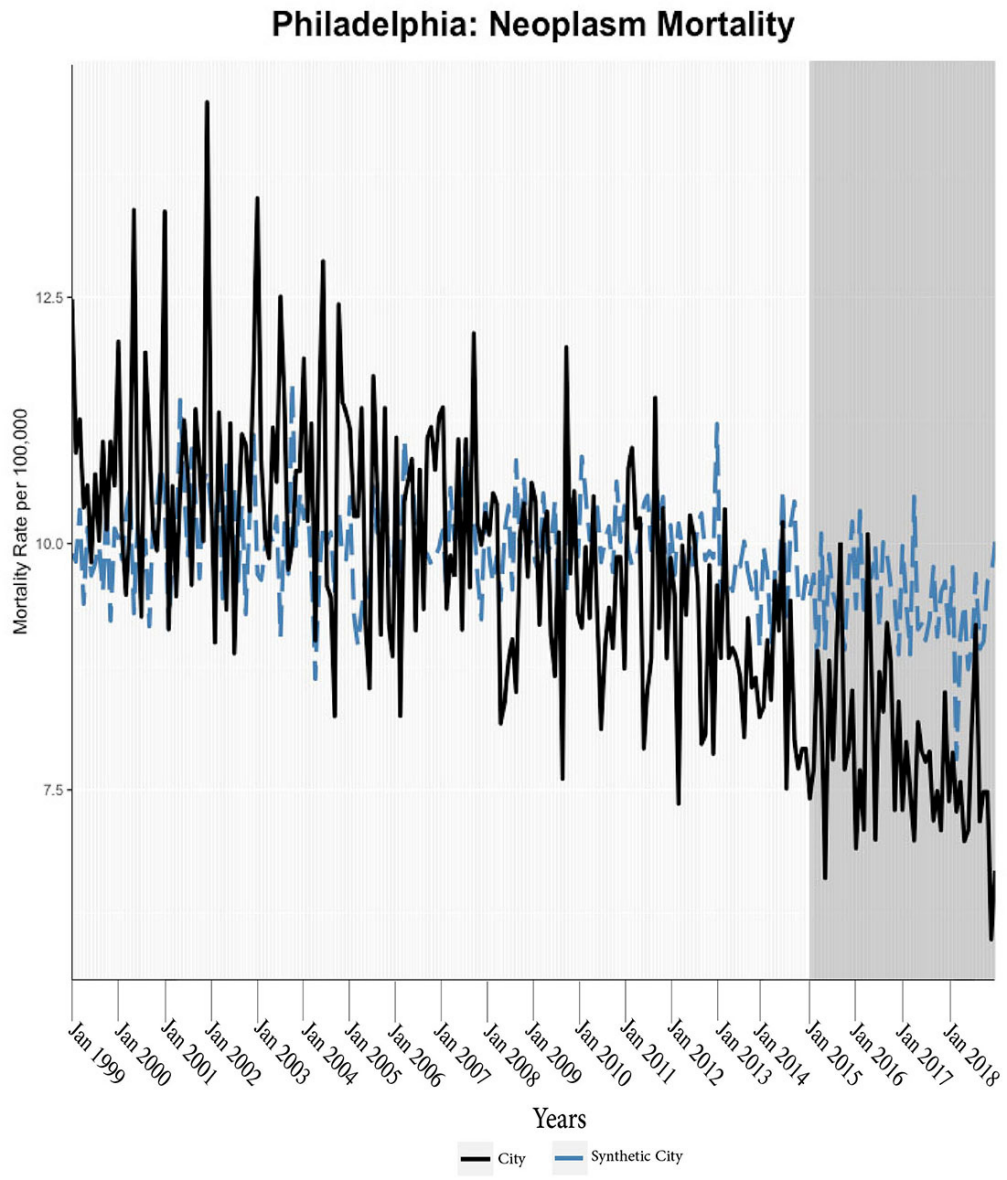
Neoplasm mortality trends: Philadelphia and synthetic control city.

**Figure 9:**
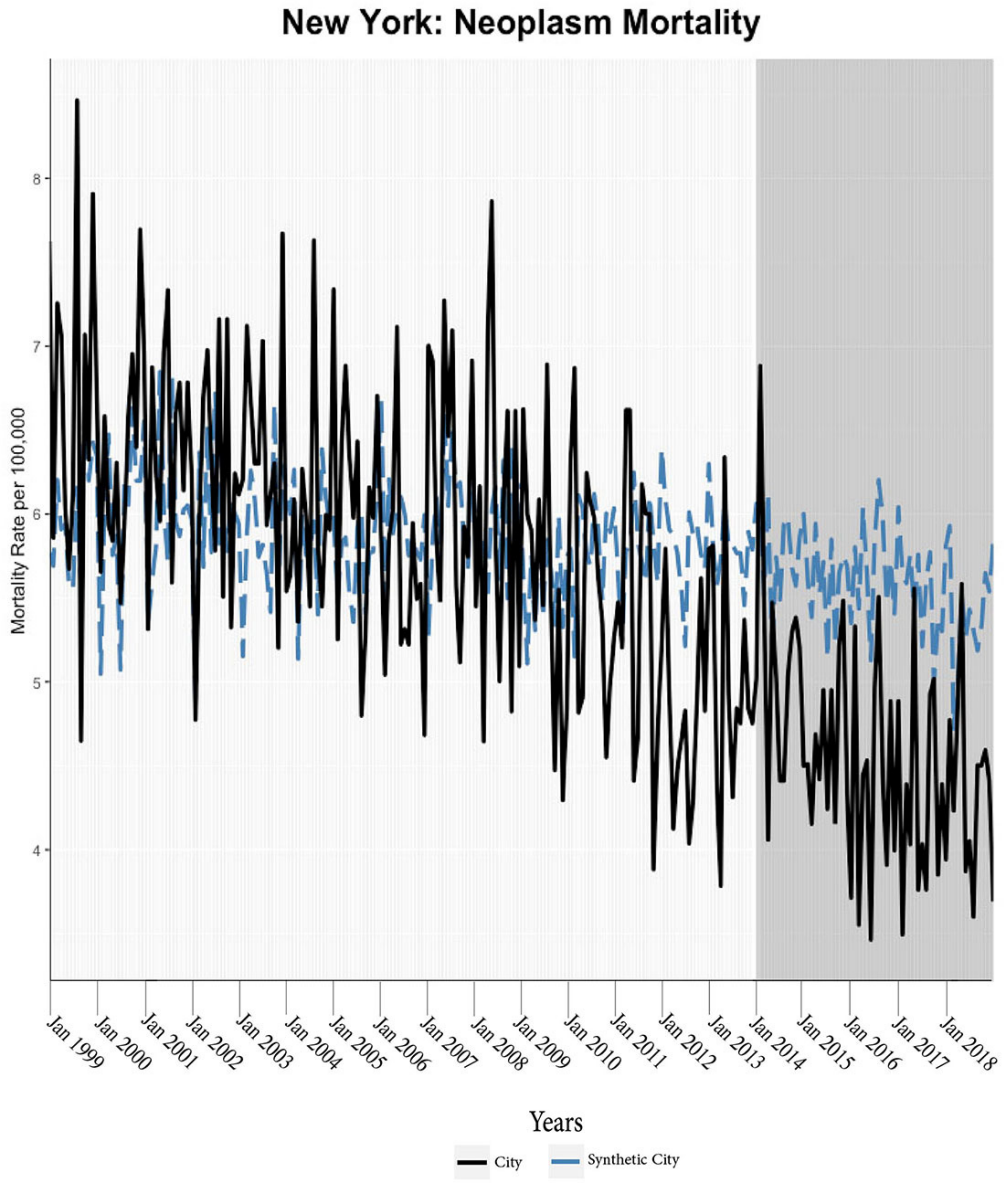
Neoplasm mortality trends: New York and synthetic control city.

**Figure 10:**
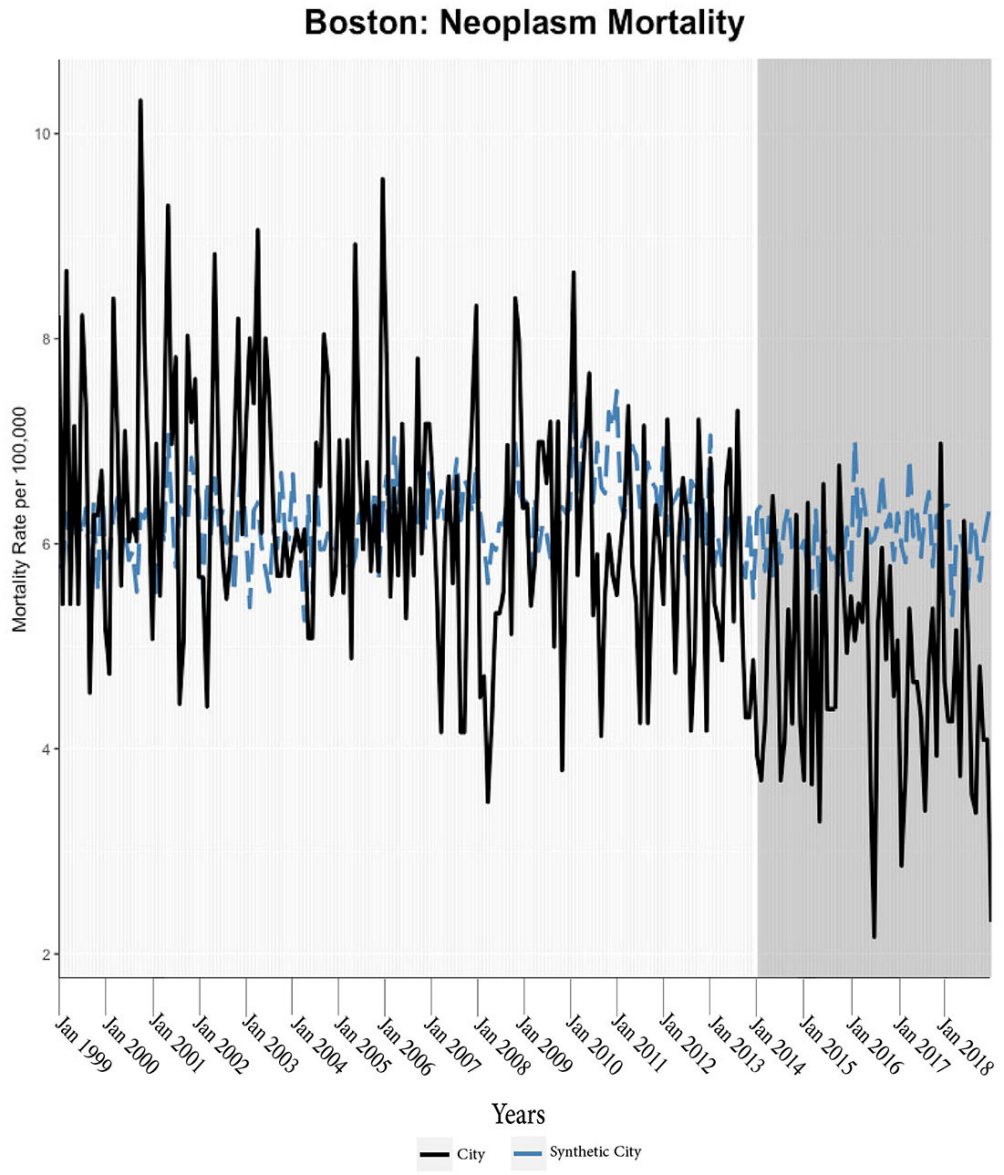
Neoplasm mortality trends: Boston and synthetic control city.

We found evidence of significant reductions in all-cause medical mortality as compared to predicted mortality had expansion not occurred in four cities, including Washington D.C., Baltimore, Philadelphia, and New York, after their 2010, 2014, 2015, and 2014 expansions of Medicaid, respectively (Figures 1–5). Similar effect sizes are seen across all cities (ATT range = −1.39 to −2.78) apart from Washington D.C., which saw a much larger decrease (ATT = −5.40). The −5.40 ATT value for Washington D.C. conveys that the average monthly neoplasm mortality rate per 100,000 was reduced by 5.40 over the post-intervention period. When assessing neoplasms, we observe a significant reduction in mortality in four cities excluding New York (Figures 5–10). Pooled across all cities, there was a significant reduction in the neoplasm (Population-Adjusted ATT = −1.37 [95% CI −2.73, −0.42]) and all-cause medical (Population-Adjusted ATT = −2.57 [95% CI −8.46, −0.58]) mortality rates but not cardiovascular (Population-Adjusted ATT = −3.79 [95% CI −24.57, 11.68]) and respiratory mortality (Population-Adjusted ATT = −0.91 [95% CI −6.99, 4.89]) (see Figures 12–22 in the Appendix).

**Figure 11:**
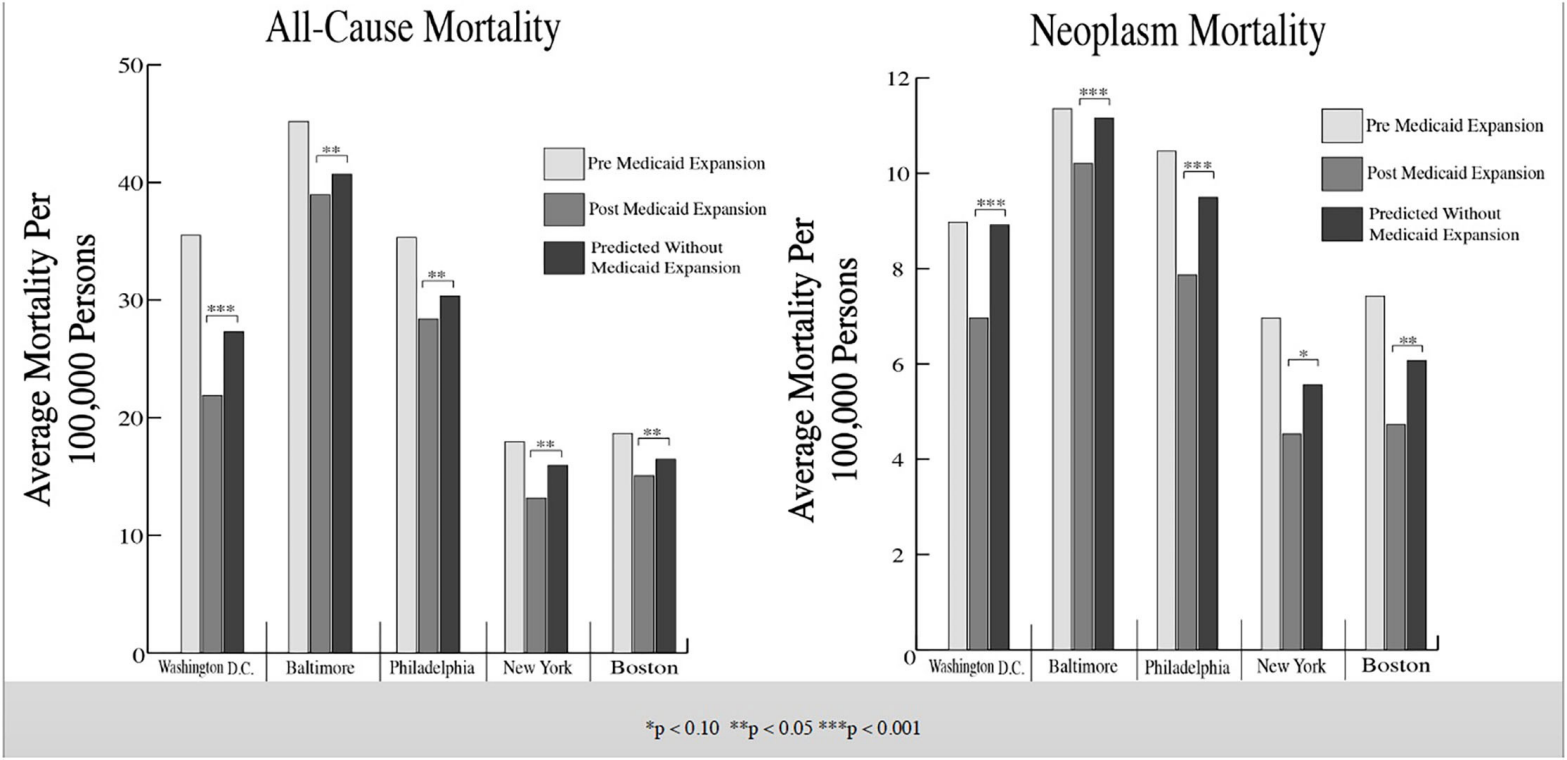
Average mortality rates among treated cities and synthetic control cities. These bar graphs represent the average mortality rate of the selected Northeastern cities. Mortality rates before and after Medicaid expansion are illustrated along with the predicted average mortality rates if Medicaid expansion had not occurred in said cities (derived by the generalized synthetic control model).

According to established practices, we discard models that have grossly mismatched pre-intervention mortality trends between the treatment and synthetic units (27); this was only observed for respiratory mortality in Baltimore, which was highly volatile. Subsequently, the treatment effect was calculated through the ATT and CTT parameters; *p*-values indicated levels of statistical significance (Table 2; Figure 11). Parameters (ATT, %ATT, or CTT), time unit of measurement (yearly or monthly), and post-intervention period length must be considered when comparing across the reported values. Overall, our synthetic control models indicate a trend of decreases in neoplasm and all-cause medical mortality rates in cities that expanded Medicaid compared to their estimated counterfactuals. Sensitivity analyses of monthly data converted to yearly data indicated that the analysis was not sensitive to yearly data. Specifically, the conversion of monthly all-cause medical and neoplasm mortality into yearly aggregates changed significant differences into insignificant differences (see Tables 1, 2 in the Appendix). The in-time placebo sensitivity analysis indicated that the false intervention time point did not result in significant change in mortality rates (see Table 3 and Figures 11–20 in the Appendix). The weighted composition of each synthetic control model in the mortality analyses is included (see Table 4 in Appendix).

**Table 2:** Treatment effects on mortality rates in treated cities.

	**ATT**	**Lower 95% CI**	**Higher 95% CI**	**ATT%^†^**	**ATT% Lower 95% CI**	**ATT% Higher 95% CI**	***p***
**Washington D.C. | 2010**							
Circulatory mortality	−14.83	−45.20	7.47	−13.62	−41.51	6.86	0.168
Respiratory mortality	2.31	−6.21	9.73	18.39	−49.41	77.43	0.658
Neoplasm mortality	−1.95	−3.04	−0.98	−21.88	−34.10	−10.99	0.002^***^
All-cause medical mortality	−5.40	−12.50	−3.34	−18.84	−43.64	−11.67	<0.001^***^
**Baltimore | 2014**							
Circulatory mortality	−7.00	−24.83	3.13	−4.35	−15.43	1.95	0.148
Respiratory mortality	1.96	−2.99	6.39	6.78	−10.33	22.08	0.482
Neoplasm mortality	−0.96	−1.90	−0.17	−8.56	−17.05	−1.53	0.01^***^
All-cause medical mortality	−1.76	−6.51	−0.28	−4.48	−16.53	−0.70	0.038^**^
**Philadelphia | 2015**							
Circulatory mortality	0.96	−13.22	9.47	0.86	−11.89	8.51	0.934
Respiratory mortality	−1.37	−5.10	1.43	−5.95	−22.21	6.22	0.336
Neoplasm mortality	−1.63	−2.73	−0.99	−17.17	−28.72	−10.39	<0.001^***^
All-cause medical mortality	−1.97	−5.92	−0.50	−6.77	−20.29	−1.71	0.018^**^
**New York | 2014**							
Circulatory mortality	−0.39	−25.30	19.57	−0.87	−56.70	43.86	0.802
Respiratory mortality	−3.00	−10.44	4.42	−26.77	−93.16	39.48	0.322
Neoplasm mortality	−1.03	−2.86	0.203	−34.46	−95.69	6.77	0.096^*^
All-cause medical mortality	−2.78	−10.65	−0.13	−18.11	−69.29	−0.86	0.04^**^
**Boston | 2014**							
Circulatory mortality	−7.33	−18.72	2.50	−15	−38.31	5.12	0.144
Respiratory mortality	−0.67	−5.19	4.68	−6.07	−47.09	42.46	0.836
Neoplasm mortality	−1.35	−2.49	−0.55	−22.17	−40.93	9.06	0.004^***^
All-cause medical mortality	−1.39	−4.53	0.09	−8.85	−28.77	0.57	0.068^*^

* *≤ 0.10*

** *≤ 0.05*

*** * ≤ 0.01*.

† *Percentage change of post-intervention mortality of Medicaid expansion city from synthetic control prediction*.

The generalized synthetic control analyses of insurance rates indicated that the observed city after Medicaid expansion were all much greater than predicted (Table 3) (see Figures 21–25 in Appendix). Most notably, the gains in Washington D.C. (ATT = 4.23%, *p* = 0.050), Baltimore (ATT = 3.23%, *p* = 0.048), and Philadelphia (ATT = 4.30% *p* = 0.046) were significantly greater than the predicted trends in the absence of Medicaid expansion. New York and Boston, two cities with already had high rates of coverage prior to Medicaid expansion and the derived treatment effects did not significantly differ from the predicted gains.

**Table 3:** Treatment effects on percentage of population insured in treated cities.

**City | Intervention year**	**ATT**	**Lower 95% CI**	**Higher 95% CI**	***p***
Washington D.C. | 2010	4.22%	0.28%	10.58%	0.048^**^
Baltimore | 2014	3.23%	0.30%	10.56%	0.046^**^
Philadelphia | 2015	4.31 %	0.24%	6.66%	0.036^**^
New York | 2014	0.86%	−13.13%	9.75%	0.860
Boston | 2014	−12.54%	−16.91%	7.80%	0.618

** * ≤ 0.05*.

## Discussion and Conclusion

Three principal conclusions can be drawn from our analysis. First, we document significant declines in all-cause medical mortality, partly driven by concurrent and significant reductions in neoplasm mortality, in most Northeastern cities following ACA Medicaid expansion. Second, while Medicaid expansion does appear to be associated with reductions in mortality rates across the cities, we observe evidence of varied effects. Third, analyses of yearly data in this study generally lacked sufficient statistical power.

Most cities experienced significant decreases in neoplasm mortality and all-cause medical mortality after Medicaid expansion as compared to the predicted rates. The potential mechanisms of this observed decline may be several. An intended function of Medicaid expansion was to decrease the uninsured rate (15, 16). While there was uptake of Medicaid coverage, much of this enrollment was moderate followed by a mild and steady increase (11). While the magnitude of increased insurance coverage may appear minor, more ill and vulnerable populations (e.g., women, African Americans, Hispanics, immigrants) gained coverage through Medicaid expansion which may explain the larger decrease in mortality observed in this study (11, 28).

New enrollees encountered reduced financial barriers which led to receiving timelier clinical and surgical care along with better ability to access prescription medicines for acute and chronic conditions (29–33). Improved self-reported and objective measures of quality as a result of Medicaid expansion accompanied increasing use of preventive care (34). In totality, Medicaid expansion reduced financial barriers and improved integration of communities in medical systems which resulted in individuals receiving more necessary and high-quality medical care (34). Additionally, Medicaid expansion has been associated with increased overall financial stability (35, 36). Greater financial stability, especially for those managing chronic conditions or suffering catastrophic events, not only allows for appropriate seeking of care but also may lead to broader health benefits. For example, families with greater financial stability are more able to achieve higher standards of living which act through material and psychosocial mechanisms to produce superior wellbeing (12, 37).

Considering neoplasm mortality, long standing research has generally considered the inability to access quality medical care as the major determinant of mortality especially in populations with lower socioeconomic status (37). Numerable studies of those with newfound access to Medicaid indicated that these individuals benefited greatly from expansion in the context of cancer care and mortality (38–42). As disease stage influences neoplasm prognosis, access to care is naturally a significant influence on neoplasm mortality (37). The longitudinal nature of oncological disease would suggest that reductions in mortality should become more profound as time passes from Medicaid expansion. Nevertheless, more proximal effects on cancer mortality due to the newfound ability to access diagnostic care and treatment have been reproduced not only in this study but also in others. Those with undiagnosed cancers were found to receive earlier screenings and diagnostic tests leading to appropriate oncological intervention (38, 42). Examining data as early as three-years after Medicaid expansion, Lin et al. found a reduction of late-stage lung cancer diagnoses and an increase in early-stage lung cancer diagnoses (41). Lung cancer as well as other aggressive forms of cancer may progress rapidly leading to appreciable mortality that can be avoided by early diagnosis as potentially related to Medicaid expansion (41).

Neoplasm mortality reductions partly, but not fully, underlie all-cause medical mortality reductions; unrelated reductions may result from other causes that are amenable to increased access; these may or may not have been captured separately in our study.

Variations in the observed effect sizes between cities may be related to distinct policy landscapes before Medicaid expansion. Boston, for example, passed the MHCR in 2006 and this already offered coverage to many near FLP. Indeed, baseline mortality rates in Boston were lower than in other cities in our study prior to Medicaid expansion. Further, extensive pre-Medicaid expansion insurance coverage and limited baseline mortality suggest that ACA Medicaid expansion impacts be minimal in Boston. Similarly, New York City had implemented state-wide low-income coverage policies for non-elderly adults prior to the formal expansion of Medicaid in 2014 (43). Specifically, the 2001 Family Health Plus program was expanded to 100% of FLP (43); this standing program may have reduced the impact of Medicaid expansion in New York City. Such characterization of these two policy landscapes is bolstered by the results of the insurance rate analysis. Specifically, New York and Boston did not observe significant gains in the insured population as compared to their predicted trajectory absent Medicaid expansion. Nevertheless, we do observe smaller and statistically insignificant gains in the insured rate after Medicaid Expansion; given the already high rates, we anticipate that only minor gains in insurance rates would have been possible, so that this limited magnitude may not achieve statistical significance.

By contrast, the remaining cities resided in states where Medicaid expansion resulted in a large expansion in their public insurance eligibility criteria. Before its early expansion of Medicaid in 2010, Washington D.C. had only adopted narrow programs beyond the federal Medicaid requirements. Washington D.C. not only expanded Medicaid early, but the district set eligibility criteria to 210% FPL, which makes this expansion the largest of any included city (44). Similarly, Pennsylvania had heavily utilized waivers to create several targeted programs but none of these programs were broadly applicable to Medicaid expansion-eligible populations. Philadelphia had similar mortality rates to Washington D.C. but the extent of Medicaid expansion was less significant (i.e., 138% FPL).

Unlike Washington D.C. and Philadelphia, the 2006 Primary Adult Care (PAC) program under the HealthChoice program in Baltimore expanded coverage (prescription, primary care, behavioral health) to childless adults at or below 116% FPL (45). Accordingly, Medicaid expansion did moderately expand Baltimore's coverage criteria from baseline. Nevertheless, Washington D.C. and Baltimore had the highest mortality rates before Medicaid expansion; this inclines both cities to larger observed effects. Medicaid expansion operated in the context of Baltimore's elevated mortality rates, higher poverty rates, and large minority populations; these factors likely promote greater Medicaid expansion effects. The aforementioned factors suggest that Medicaid expansion might be most efficacious in Washington D.C., Baltimore and Philadelphia. The insurance rate models found significant increases in the insured percentage of said cities as compared to those trends predicted in the absence of Medicaid expansion. This provides evidence that Medicaid expansion may have increased insurance rates significantly.

Importantly, our findings diverge from state-wide analyses, in that, the magnitude of Medicaid expansion impact was larger in urban areas than in states as a whole. We attribute this to multiple factors. First, the concentration of health issues in urban areas may be more able to capture the health effects of Medicaid expansion (21). Second, greater proximity to medical care in urban environments, unlike rural areas, suggests that financial access is a stronger variable in determining healthcare access (46). With respect to Medicaid expansion specifically, previous studies have shown that medical utilization starkly increased in states after the introduction of Medicaid expansion (28). Such observed increases in utilization along with broader increases in physician supply and particularly high densities of healthcare resources in large cities strongly suggest that the increased access to care was achieved through Medicaid coverage (28, 46). This further suggests that lesser gains in urban coverage, as compared to rural populations, can be potent in improving population health in urban settings (47).

Certain limitations should be considered. Sensitivity analyses indicated that yearly data were unable to derive similarly statistically significant treatment effects as compared to monthly data. This may be explained by differences in granularity reducing study power, seasonal trends, etc. Thus, it is likely that the yearly circulatory and respiratory mortality data used are inadequate to capture treatment effects. Otherwise, mortality is an extreme marker of population health not fully representative of the total effects of Medicaid expansion. Considering trend volatility, dissimilar baseline mortality, different intervention sizes, and the fundamental limitations in the GSC, lack of findings should not be considered as precluding effects on certain types of mortality (e.g., respiratory).

Like most other Medicaid expansion studies, this study fundamentally compares mortality rates between non-ME and Medicaid expansion states where these groups may experience unique confounding influences given the ecological nature of the data. For instance, city specific events related to police brutality, weather events, civil unrest, etc., may be lay the ground for specific forms of medical-related mortality *via* more diffuse factors (e.g., stress, slow medical emergency response times). Nevertheless, these effects may not strongly affect the sum magnitude of the observed mortality and would likely only dampen observed degree effects attributed to Medicaid expansion in the present study. The use of only urban populations, numerous independent relevant covariates, and the generalized synthetic control method allow this study to limit the influence of observed and unobserved confounding unlike most previous studies of Medicaid expansion. Regardless, overfitting is a concern in synthetic control models and while the GSC model does improve upon overfitting issues found in the traditional synthetic control methods *via* the use of semiparametric estimation and cross-validation schema, the risk of overfitting remains (25).

Unlike most literature, the county-level nature of this analysis limits the number of individuals available for study. As such, separate analyses of older sub-populations were inviable given the limited size of such a sub-population and the risk of stochastic variability biasing the model. Nevertheless, the inclusion of younger and healthier populations in the presented results more likely underestimates the treatment effects of Medicaid expansion (28). Similarly, the nature of the data utilized county-wide covariates, as such analyses of mortality rates in subsets of particular covariate categories (e.g., education-level) was unable to be conducted. Further studies are required to determine whether the observed effects of Medicaid expansion can be generalized to other cities without Medicaid expansion. To this point, each city's results must be considered given the unique treatment intensity and policy history characterized above.

Significant reductions in multiple forms of urban mortality were attributed to Medicaid expansion. The degree of effects was seemingly related to baseline mortality rates, prior expansion status, and the magnitude of Medicaid expansion. Our study indicates that Medicaid expansion saved lives in the included urban settings.

## Data Availability Statement

The original contributions presented in the study are included in the article/Supplementary Material, further inquiries can be directed to the corresponding author.

## Author Contributions

CA designed and conducted the initial gathering of data, organization, analysis, and organized the findings and subsequently put the information in a manuscript format. PP was involved in the data analysis, figure making, and the writing of the manuscript. SA was involved in the organization and the writing of the manuscript. All authors contributed to the article and approved the submitted version.

## Conflict of Interest

The authors declare that the research was conducted in the absence of any commercial or financial relationships that could be construed as a potential conflict of interest.

## Publisher's Note

All claims expressed in this article are solely those of the authors and do not necessarily represent those of their affiliated organizations, or those of the publisher, the editors and the reviewers. Any product that may be evaluated in this article, or claim that may be made by its manufacturer, is not guaranteed or endorsed by the publisher.

## References

[1] Blumenthal D Abrams M Nuzum R. The affordable care act at 5 years. N Engl J Med. (2015) 372:2451–8. 10.1056/NEJMhpr1503614 25946142

[2] Jacobs LR Callaghan T. Why states expand medicaid: Party, resources, and history. J Health Polit Policy Law. (2013) 38:1023–50. 10.1215/03616878-2334889 23794741

[3] Kaestner R Garrett B Chen J Gangopadhyaya A Fleming C. Effects of ACA medicaid expansions on health insurance coverage and labor supply. J Policy Anal Manage. (2017) 36:608–42. 10.1002/pam.21993 28653821

[4] Isola S Reddivari AKR. Affordable Care Act. [Updated 2021 Jul 15]. In: *StatPearls [Internet]*. Treasure Island, FL: StatPearls Publishing (2021). 31747174

[5] Goodman-Bacon A. Public insurance and mortality: evidence from medicaid implementation. J Polit Econ. (2018) 126:216–62. 10.1086/695528

[6] Currie J Gruber J. Health insurance eligibility, utilization of medical care, and child health. Q J Econ. (1996) 111:431–66. 10.2307/29466842430504

[7] Howell EM. The impact of the Medicaid expansions for pregnant women: a synthesis of the evidence. Med Care Res Rev. (2001) 58:3–30. 10.1177/107755870105800101 11236231

[8] Sommers BD Long SK Baicker K. Changes in mortality after Massachusetts health care reform: a quasi-experimental study. Ann Intern Med. (2014) 160:585–93. 10.7326/M13-2275 24798521

[9] Powell D. Imperfect Synthetic Controls: Did the Massachusetts Health Care Reform Save Lives? (2018).

[10] Sommers BD. State medicaid expansions and mortality, revisited: a cost-benefit analysis. Am J Health Econ. (2017) 3:392–421. 10.1162/ajhe_a_00080

[11] Finkelstein A Taubman S Wright B Bernstein M Gruber J Newhouse JP. The Oregon health insurance experiment: evidence from the first year. Q J Econ. (2012) 127:1057–106. 10.1093/qje/qjs020 23293397 PMC3535298

[12] Borgschulte M Vogler J. Did the ACA medicaid expansion save lives? J Health Econ. (2020) 72:102333. 10.1016/j.jhealeco.2020.102333 32592924

[13] Black B Hollingsworth A Nunes L Simon K. The Effect of Health Insurance on Mortality: Power Analysis and What We Can Learn from the Affordable Care Act Coverage Expansions. National Bureau of Economic Research (2019). p. 0898–2937.

[14] Miller S Altekruse S Johnson N Wherry LR. Medicaid and Mortality: New Evidence from Linked Survey and Administrative Data. National Bureau of Economic Research (2019). p. 0898–2937.

[15] Bhatt CB Beck-Sagué CM. Medicaid expansion and infant mortality in the United States. Am J Pubicl Health. (2018) 108:565–67. 10.2105/AJPH.2017.304218 29346003 PMC5844390

[16] Eliason EL. Adoption of Medicaid Is Associated with Lower Maternal Mortality. Women's Health Issues (2020). 30:147–52. 10.1016/j.whi.2020.01.005 32111417

[17] Khatana SAM Bhatla A Nathan AS Giri J Shen C Kazi DS. Association of Medicaid expansion with cardiovascular mortality. JAMA Cardiol. (2019) 4:671–79. 10.1001/jamacardio.2019.1651 31166575 PMC6552110

[18] Swaminathan S Sommers BD Thorsness R Mehrotra R Lee Y Trivedi AN. Association of Medicaid expansion with 1-year mortality among patients with end-stage renal disease. JAMA. (2018) 320:2242–50. 10.1001/jama.2018.16504 30422251 PMC6417808

[19] Freudenberg N. Time for a national agenda to improve the health of urban populations. Am J Publ Health. (2000) 90:837. 10.2105/AJPH.90.6.837 10846496 PMC1446275

[20] Geronimus AT. To mitigate, resist, or undo: addressing structural influences on the health of urban populations. Am J Public Health. (2000) 90:867. 10.2105/AJPH.90.6.867 10846503 PMC1446247

[21] Vlahov D Freudenberg N Proietti F Ompad D Quinn A Nandi V. Urban as a determinant of health. J Urban Health. (2007) 84:16–26. 10.1007/s11524-007-9169-3 17356903 PMC1891649

[22] Ompad DC Galea S Caiaffa WT Vlahov D. Social determinants of the health of urban populations: methodologic considerations. J Urban Health. (2007) 84:42–53. 10.1007/s11524-007-9168-4 17458704 PMC1891644

[23] Vafaei A Rosenberg MW Pickett W. Relationships between income inequality and health: a study on rural and urban regions of Canada. Rural Remote Health. (2010) 10:1430. 20504049

[24] Day JC. Rates of uninsured fall in rural counties, remain higher than Urban counties. Census.gov. (2021). Available online at: https://www.census.gov/library/stories/2019/04/health-insurance-rural-america.html (accessed November 3, 2021).

[25] Xu Y. Generalized synthetic control method: causal inference with interactive fixed effects models. Polit Anal. (2017) 25:57–76. 10.1017/pan.2016.230886898

[26] O'Neill S Kreif N Sutton M Grieve R. A comparison of methods for health policy evaluation with controlled pre-post designs. Health Serv Res. (2020) 55:328–38. 10.1111/1475-6773.13274 32052455 PMC7080394

[27] Abadie A Diamond A Hainmueller J. Comparative politics and the synthetic control method. Am J Polit Sci. (2015) 59:495–510. 10.1111/ajps.1211630443504

[28] Sommers BD Blendon RJ Orav EJ Epstein AM. Changes in utilization and health among low-income adults after Medicaid expansion or expanded private insurance. JAMA Intern Med. (2016) 176:1501–9. 10.1001/jamainternmed.2016.4419 27532694

[29] Chou SC Gondi S Weiner SG Schuur JD Sommers BD. Medicaid expansion reduced emergency department visits by low-income adults due to barriers to outpatient care. Med Care. (2020) 58:511–8. 10.1097/MLR.0000000000001305 32000172

[30] Adamson BJ Cohen AB Estevez M Magee K Williams E Gross CP. Affordable Care Act (ACA) medicaid expansion impact on racial disparities in time to cancer treatment. Am Soc Clin Oncol. (2019) 27. 10.1200/JCO.2019.37.18_suppl.LBA1

[31] Mahendraratnam N Dusetzina SB Farley JF. Prescription drug utilization and reimbursement increased following state Medicaid expansion in 2014. J Manag Care Spec Pharm. (2017) 23:355–63. 10.18553/jmcp.2017.23.3.355 28230452 PMC10398028

[32] Loehrer AP Chang DC Scott JW Hutter MM Patel VI Lee JE. Association of the affordable care act medicaid expansion with access to and quality of care for surgical conditions. JAMA Surg. (2018) 153:e175568. 10.1001/jamasurg.2017.5568 29365029 PMC5885934

[33] Lin S Brasel KJ Chakraborty O Glied SA. Association between medicaid expansion and the use of outpatient general surgical care among US adults in multiple states. JAMA Surg. (2020) 155:1058–66. 10.1001/jamasurg.2020.2959 32822464 PMC7439213

[34] Cole MB Galárraga O Wilson IB Wright B Trivedi AN. At federally funded health centers, medicaid expansion was associated with improved quality of care. Health Aff . (2017) 36:40–8. 10.1377/hlthaff.2016.0804 28069845

[35] Allen HL Eliason E Zewde N Gross T. Can medicaid expansion prevent housing evictions? Health Aff . (2019) 38:1451–7. 10.1377/hlthaff.2018.05071 31479379

[36] Hu L Kaestner R Mazumder B Miller S Wong A. The effect of the affordable care act Medicaid expansions on financial wellbeing. J Public Econ. (2018) 163:99–112. 10.1016/j.jpubeco.2018.04.009 30393411 PMC6208351

[37] Wherry LR Miller S Kaestner R Meyer BD. Childhood medicaid coverage and later-life health care utilization. Rev Econ Stat. (2018) 100:287–302. 10.1162/REST_a_00677 31057184 PMC6497159

[38] Takvorian SU Oganisian A Mamtani R Nandi M Shulman LN Bekelman JE. Association of medicaid expansion under the affordable care act with insurance status, cancer stage, and timely treatment among patients with breast, colon, and lung cancer. JAMA Netw Open. (2020) 3:e1921653. 10.1001/jamanetworkopen.2019.21653 32074294 PMC12549135

[39] Fu S Rose L Knowlton L. The affordable care act and insurance status, stage, and timely treatment among patients with cancer: what are the possible effects? JAMA Netw Open. (2020) 3:e192169. 10.1001/jamanetworkopen.2019.21690 32074284

[40] Dawes AJ Louie R Nguyen DK Maggard-Gibbons M Parikh P Ettner SL. The impact of continuous medicaid enrollment on diagnosis, treatment, and survival in six surgical cancers. Health Serv Res. (2014) 49:1787–811. 10.1111/1475-6773.12237 25256223 PMC4254125

[41] Lin L Soni A Sabik LM Drake C. Early- and late-stage cancer diagnosis under 3 years of medicaid expansion. Am J Prev Med. (2021) 60:104–9. 10.1016/j.amepre.2020.06.020 33191064 PMC7750288

[42] Hendryx M Luo J. Increased cancer screening for low-income adults under the affordable care act medicaid expansion. Med Care. (2018) 56:944–949. 10.1097/MLR.0000000000000984 30199428

[43] Black LI Schiller JS. State variation in health care service utilization: United States, 2014. NCHS Data Brief . (2016) 245:1–8. 27227489

[44] Sommers BD Arntson E Kenney GM Epstein AM. Lessons from early medicaid expansions under health reform: interviews with medicaid officials. Medicare Medicaid Res Rev. (2013) 3:mmrr.003.04.a02. 10.5600/mmrr.003.04.a02 24834369 PMC4015416

[45] Pollak AN Steffen CB. Study of Mortality Rates of African American Infants and Infants in Rural Areas (n.d.). Available online at: https://mhcc.maryland.gov/mhcc/pages/home/workgroups/documents/african_american_study/DRAFTRPT4WRKGRP82719.pdf (accessed November, 2021).

[46] Hartley D. Rural health disparities, population health, and rural culture. Am J Public Health. (2004) 94:1675–8. 10.2105/AJPH.94.10.1675 15451729 PMC1448513

[47] Soni A Hendryx M Simon K. Medicaid expansion under the affordable care act and insurance coverage in rural and urban areas. J Rural Health. (2017) 33:217–26. 10.1111/jrh.12234 28114726

