# Supplementary material for: A Quasi-Experimental Study of Medicaid Expansion and Urban Mortality in the American Northeast
Source: Front Public Health. 2021 Nov 17;9:707907. doi: 10.3389/fpubh.2021.707907 (PMC8637894; doi:10.3389/fpubh.2021.707907)
Supplement: Supplementary file 1 [file Data_Sheet_1.docx]

Figures 1-10
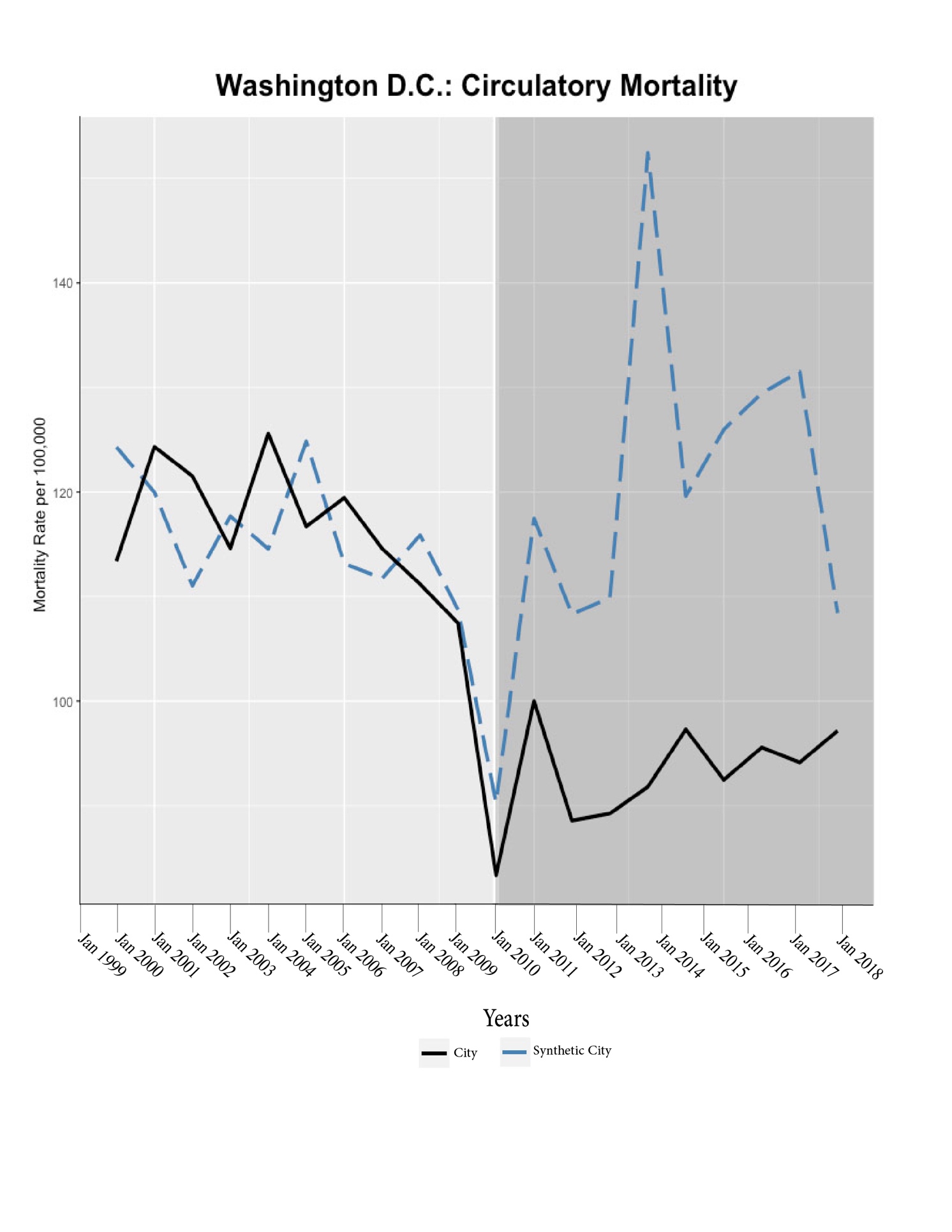

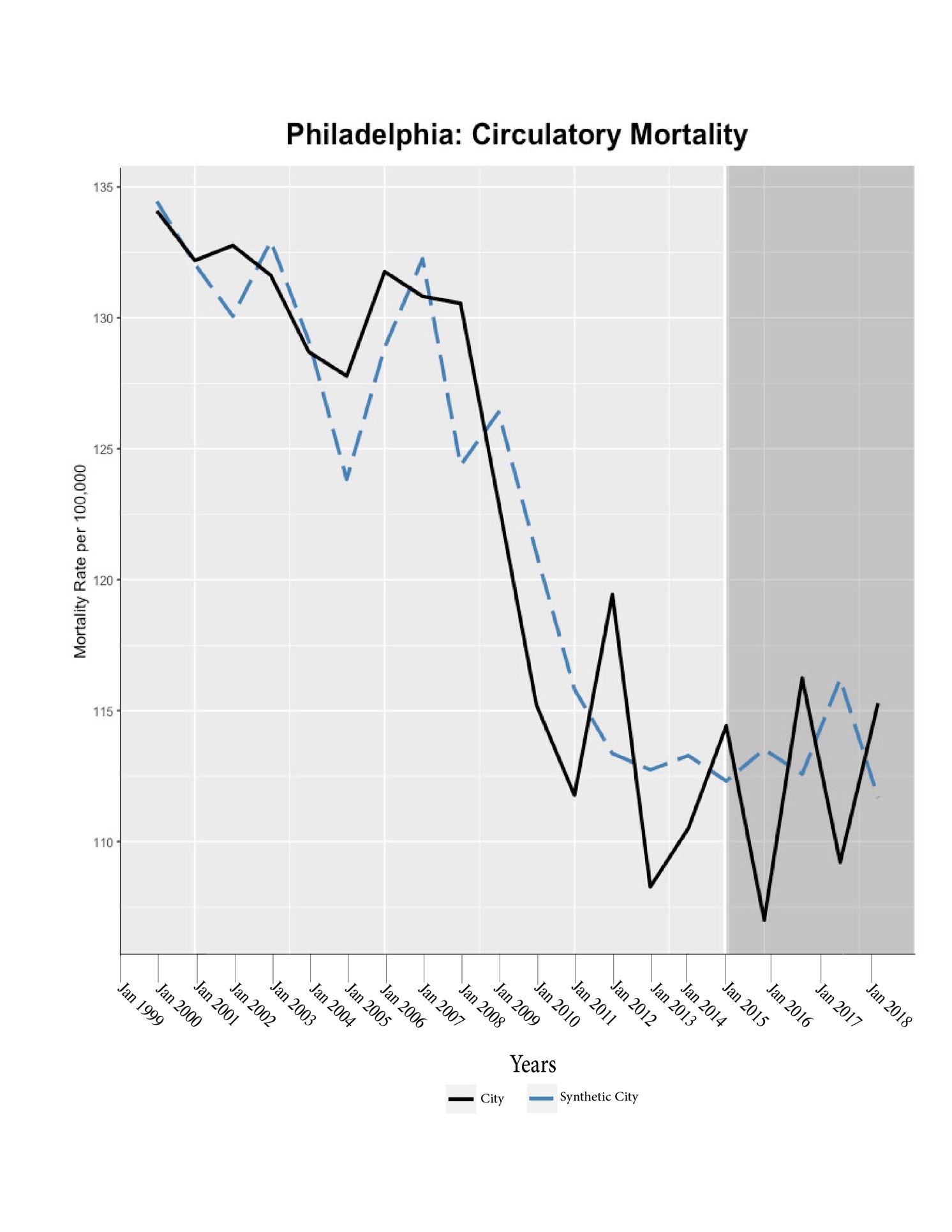

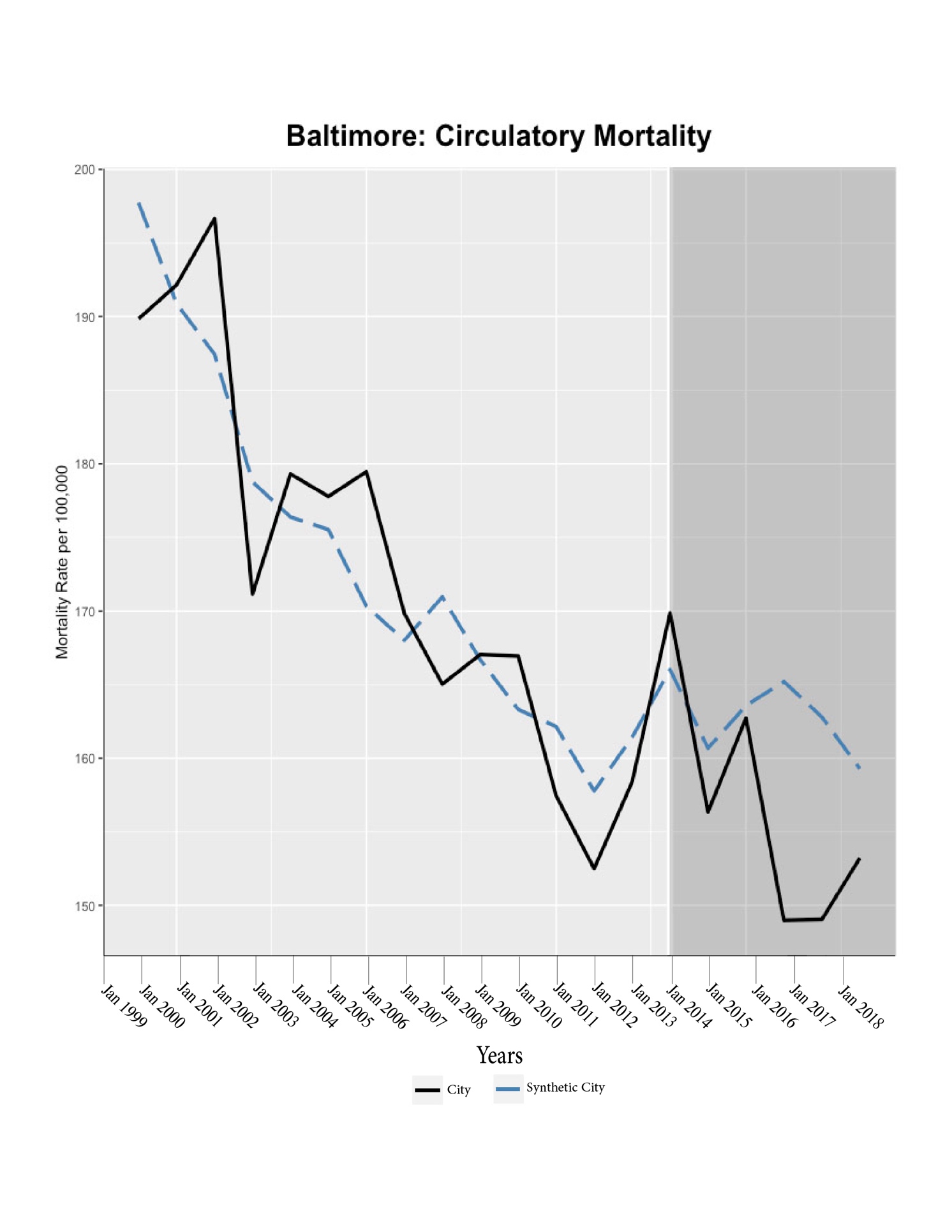

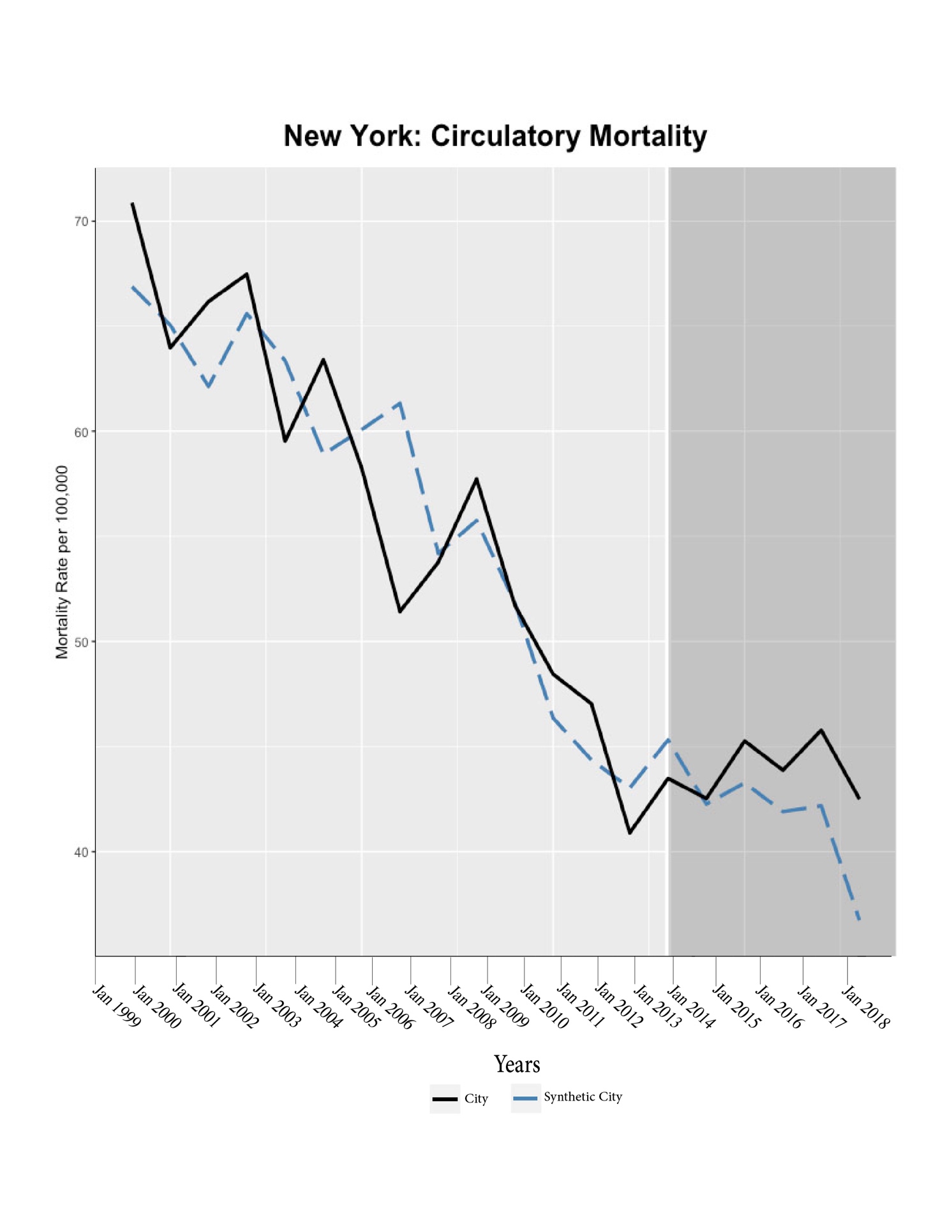

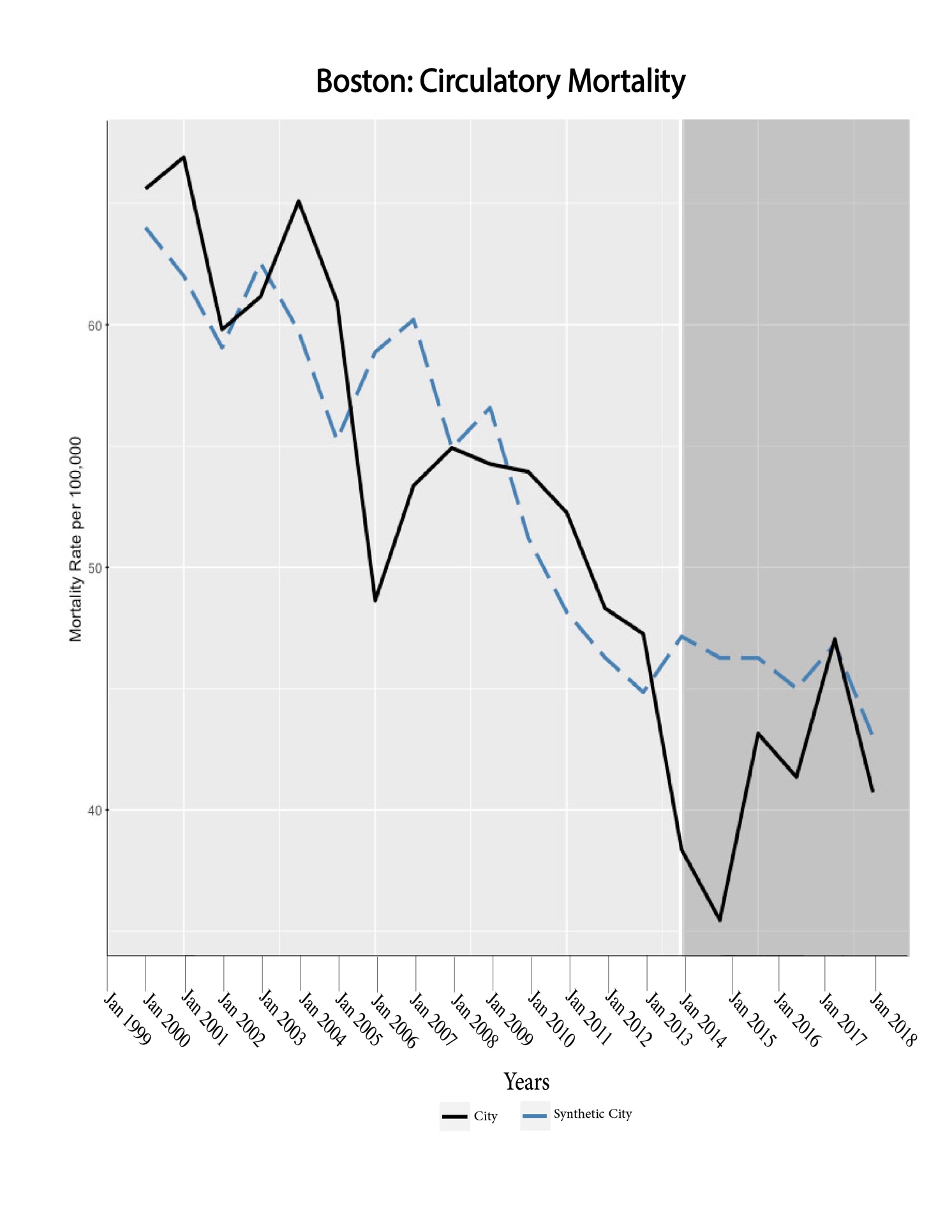

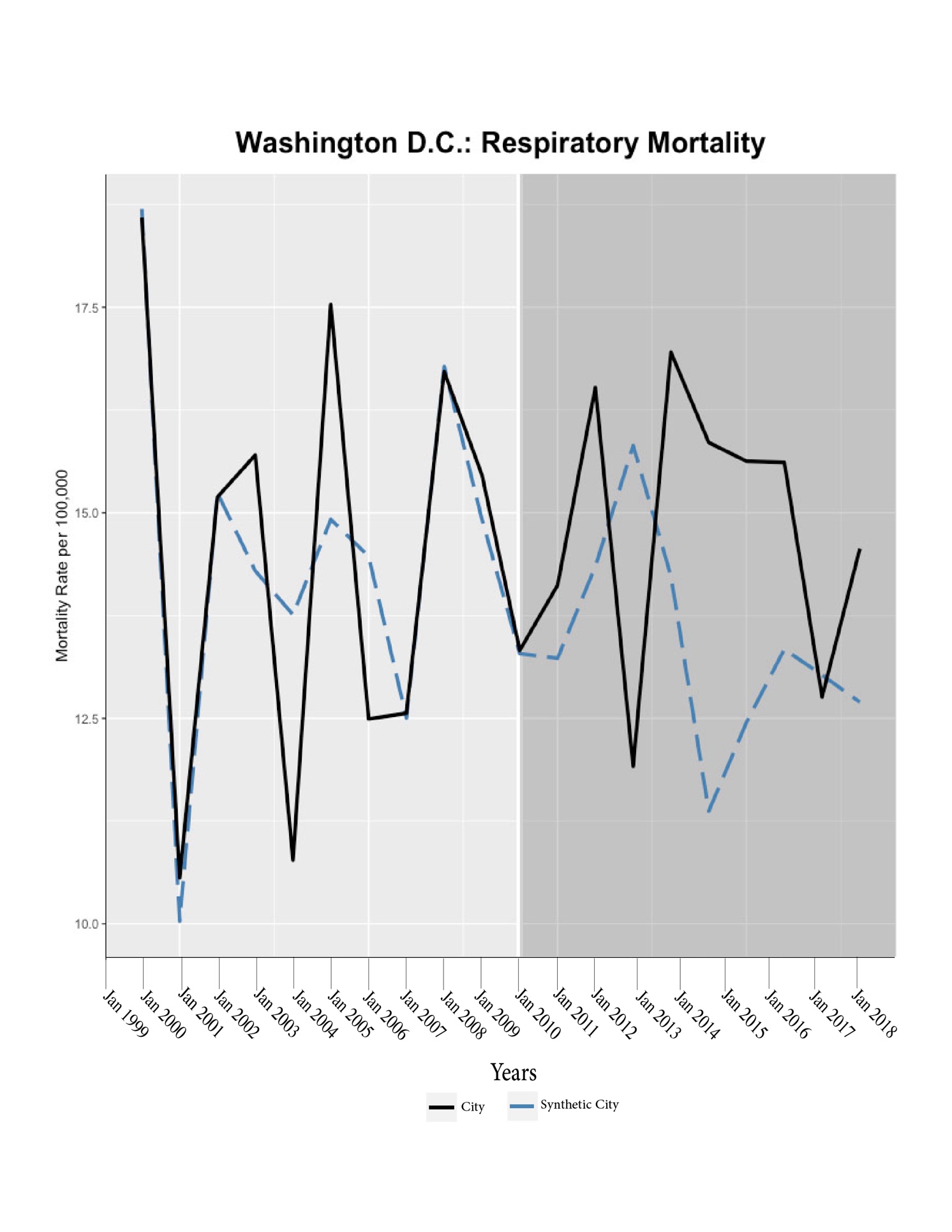

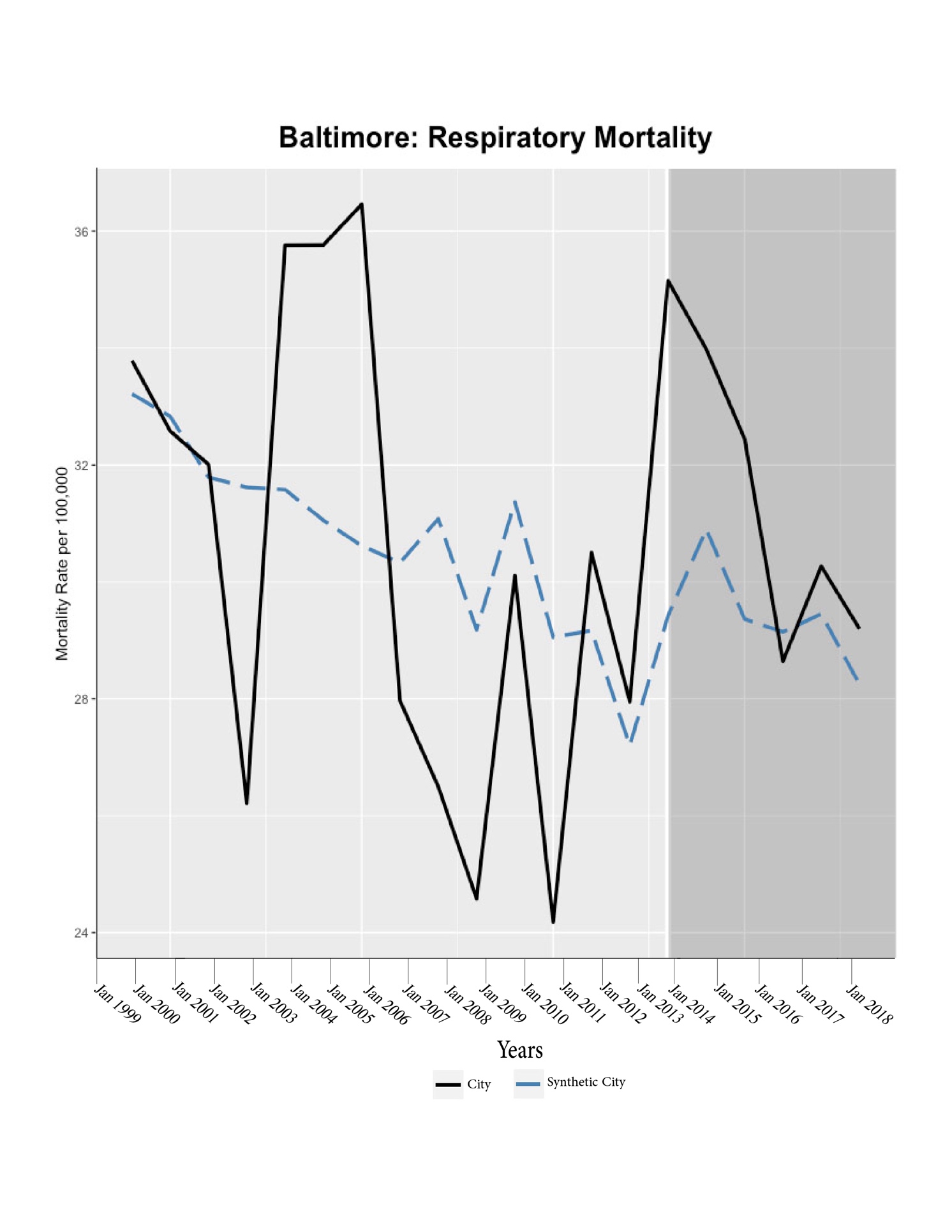

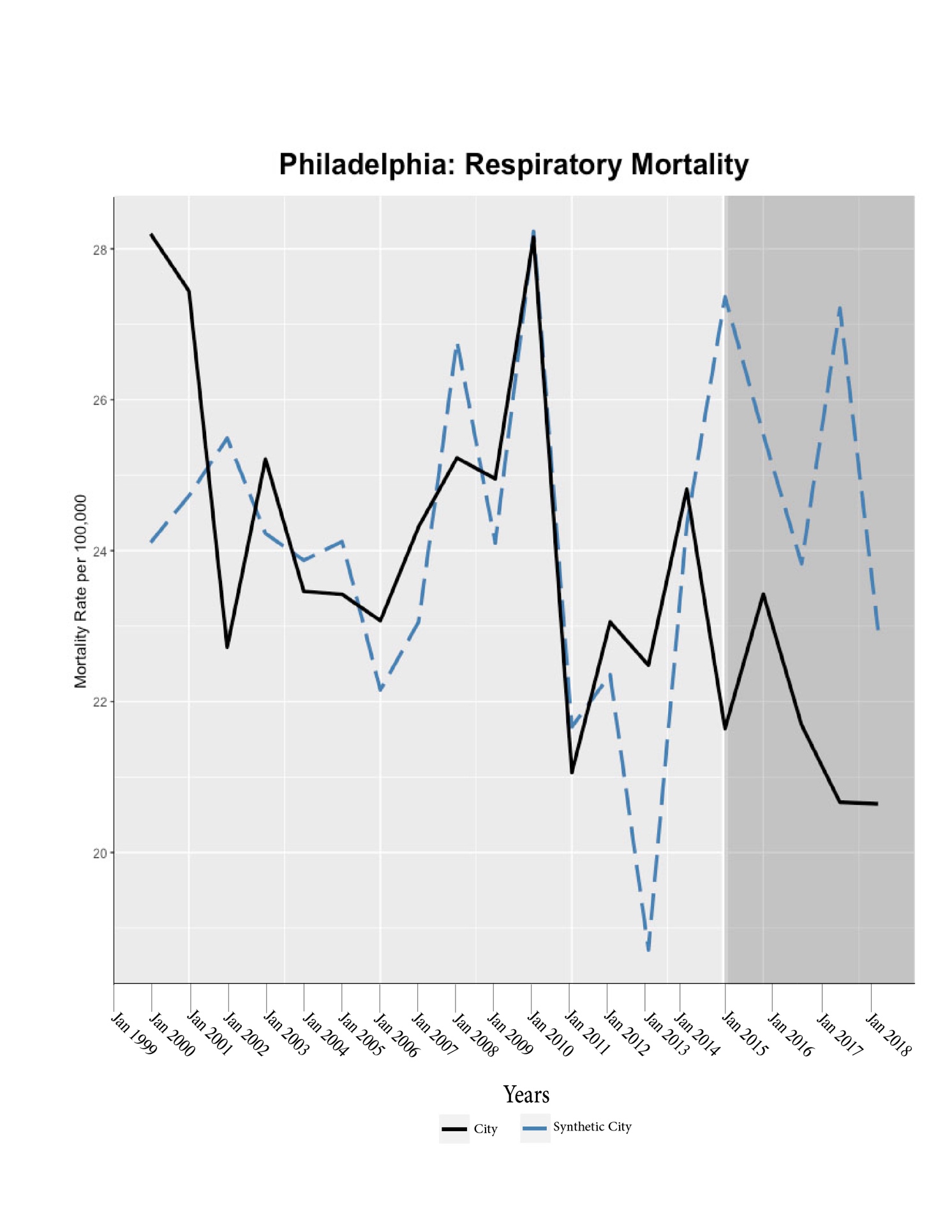

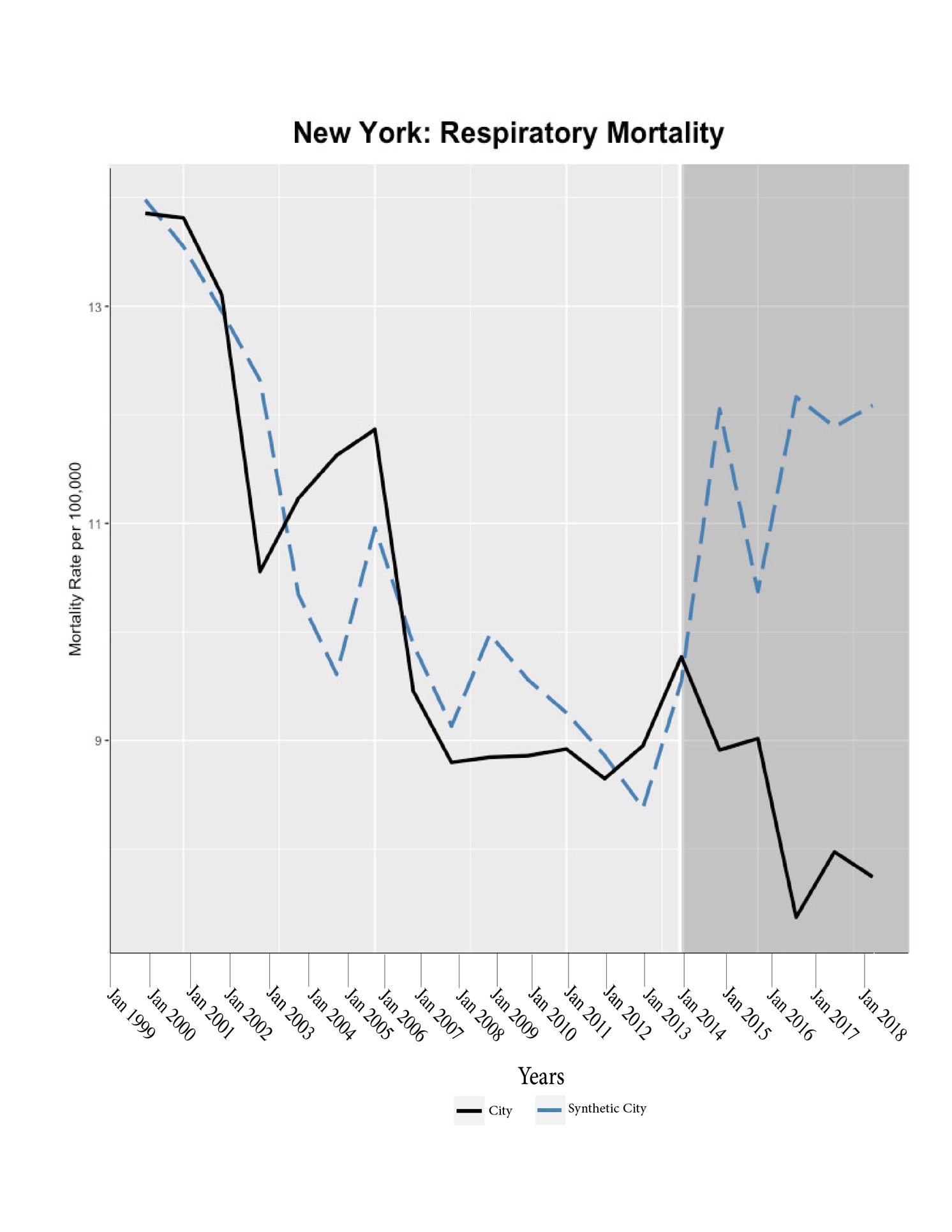

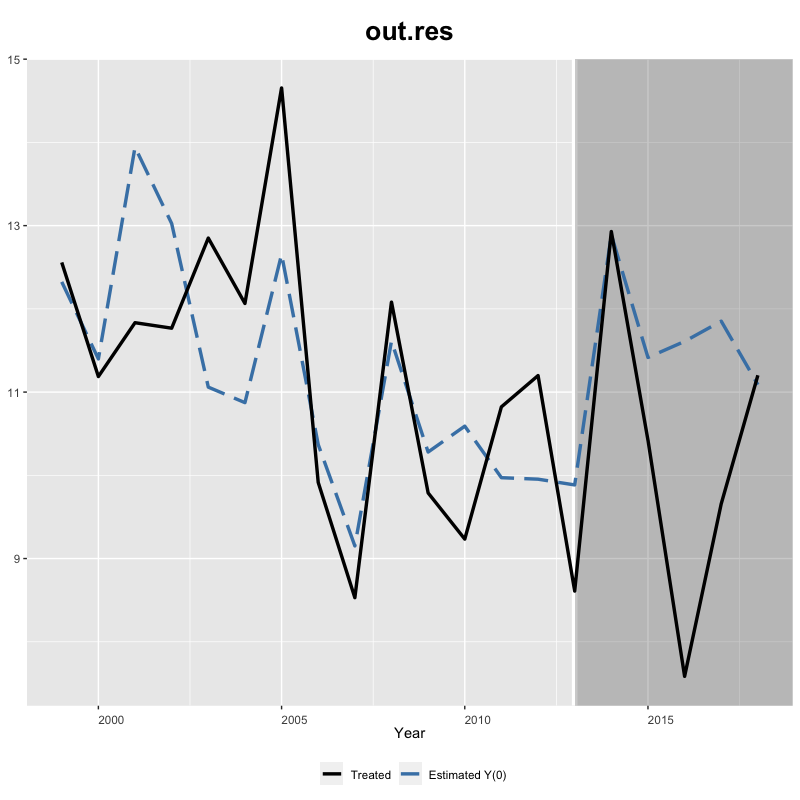

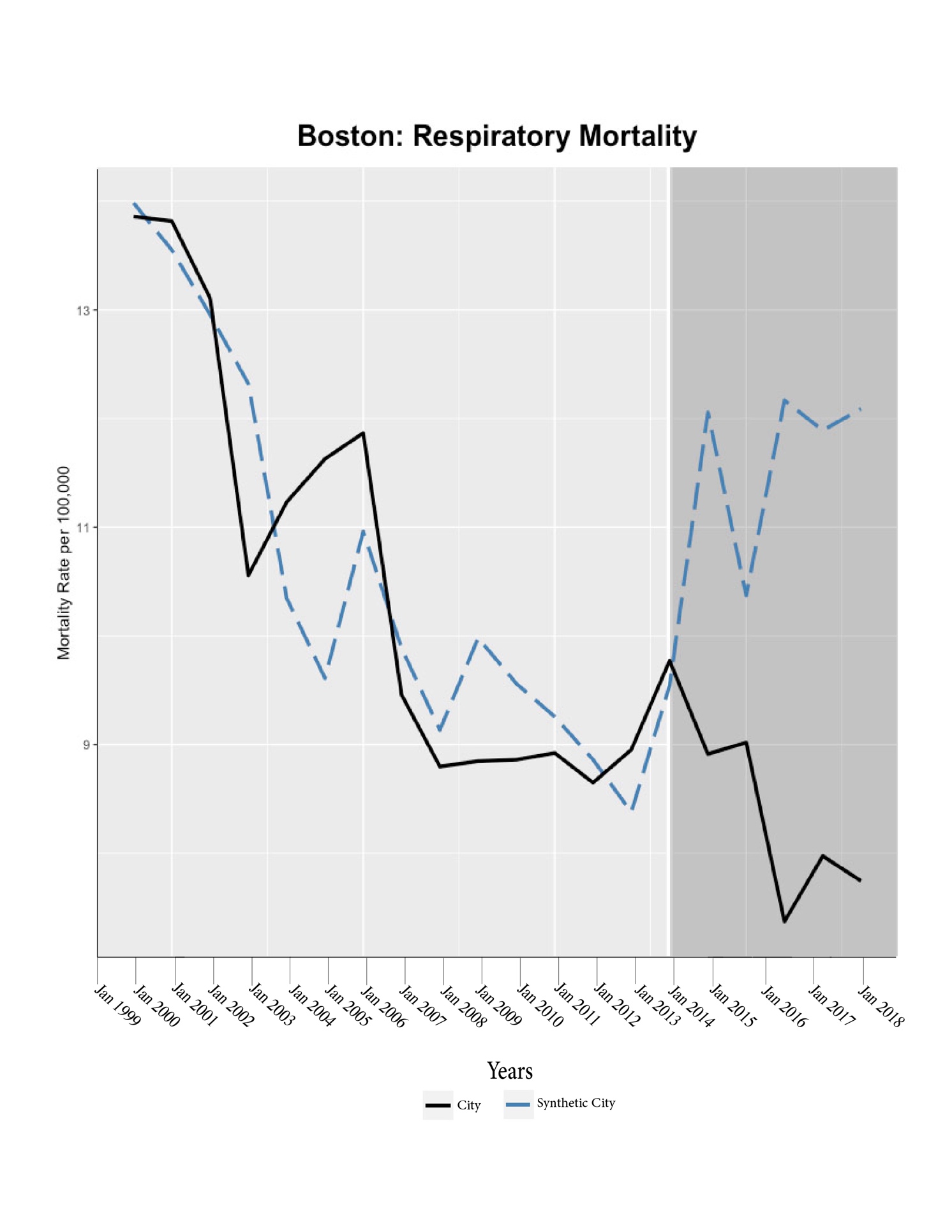


Legend: The blue dotted lines represent the generalized synthetic control prediction (synthetic city) of mortality rates while the solid black line is the observed mortality rate of the Medicaid expansion city. The vertical axis of the graphs represents the per 100,000-person mortality rates. The horizontal axis of the graphs represents time units (i.e. the first month of the year). The darker grey graph areas correlate with the start and duration of Medicaid expansion in the respective city.

Figure 11-20
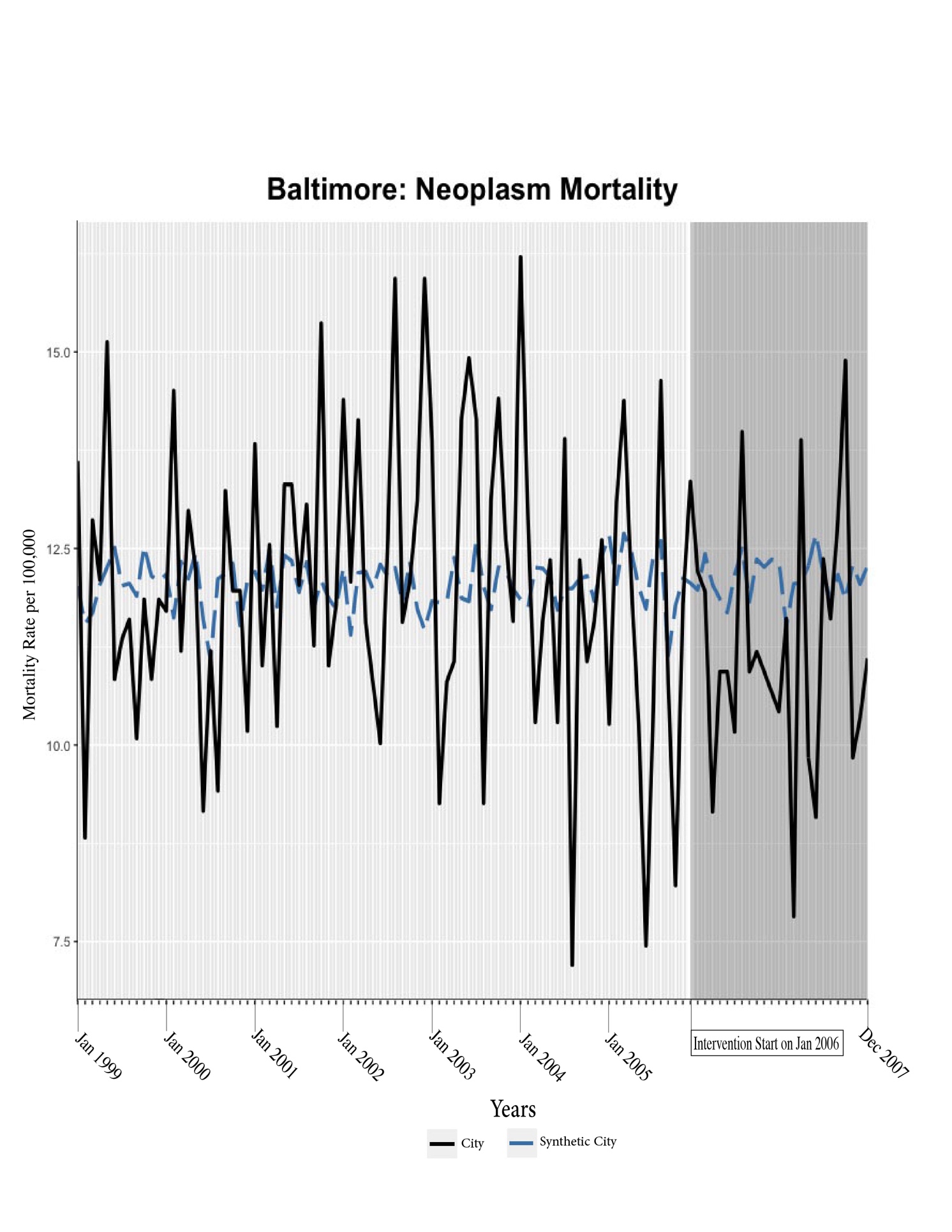

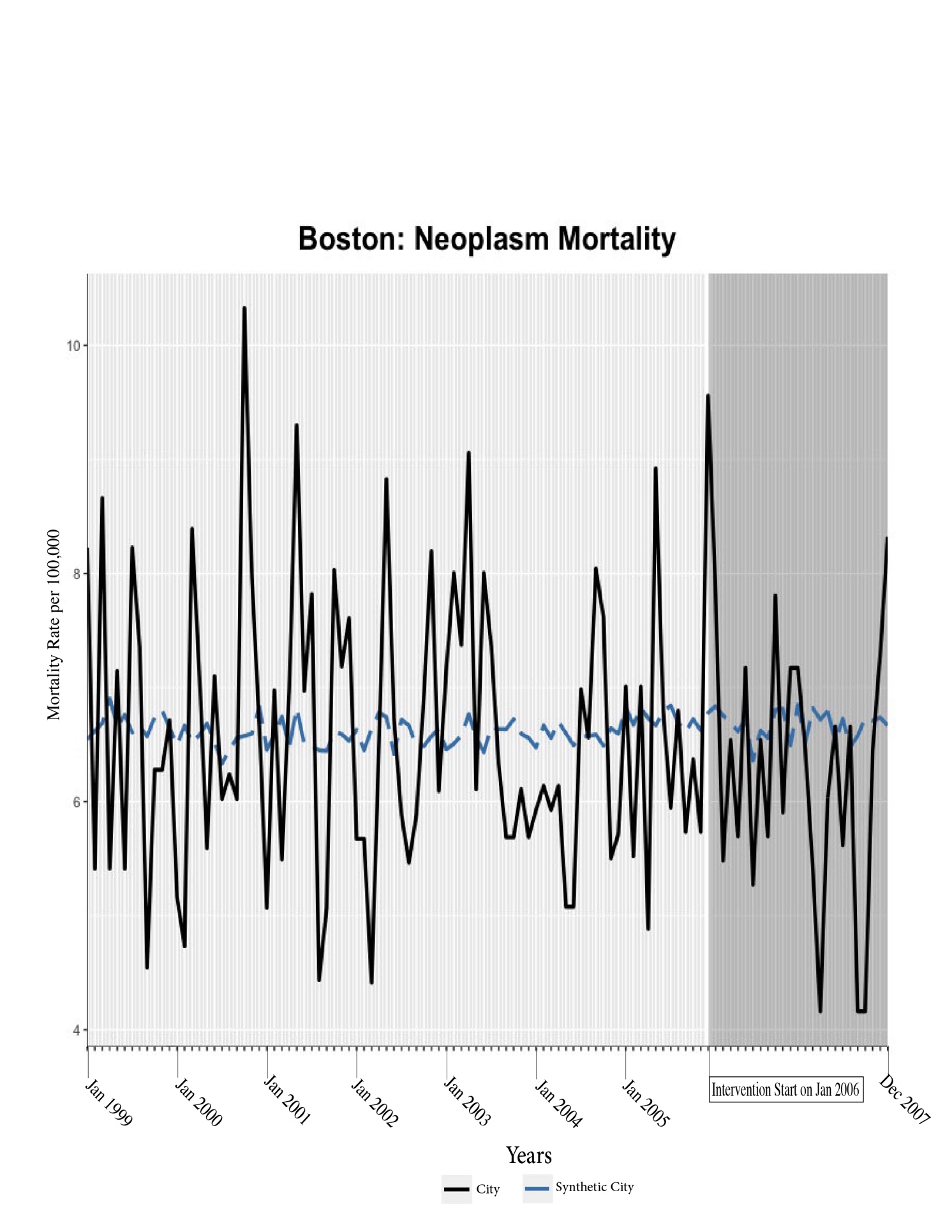

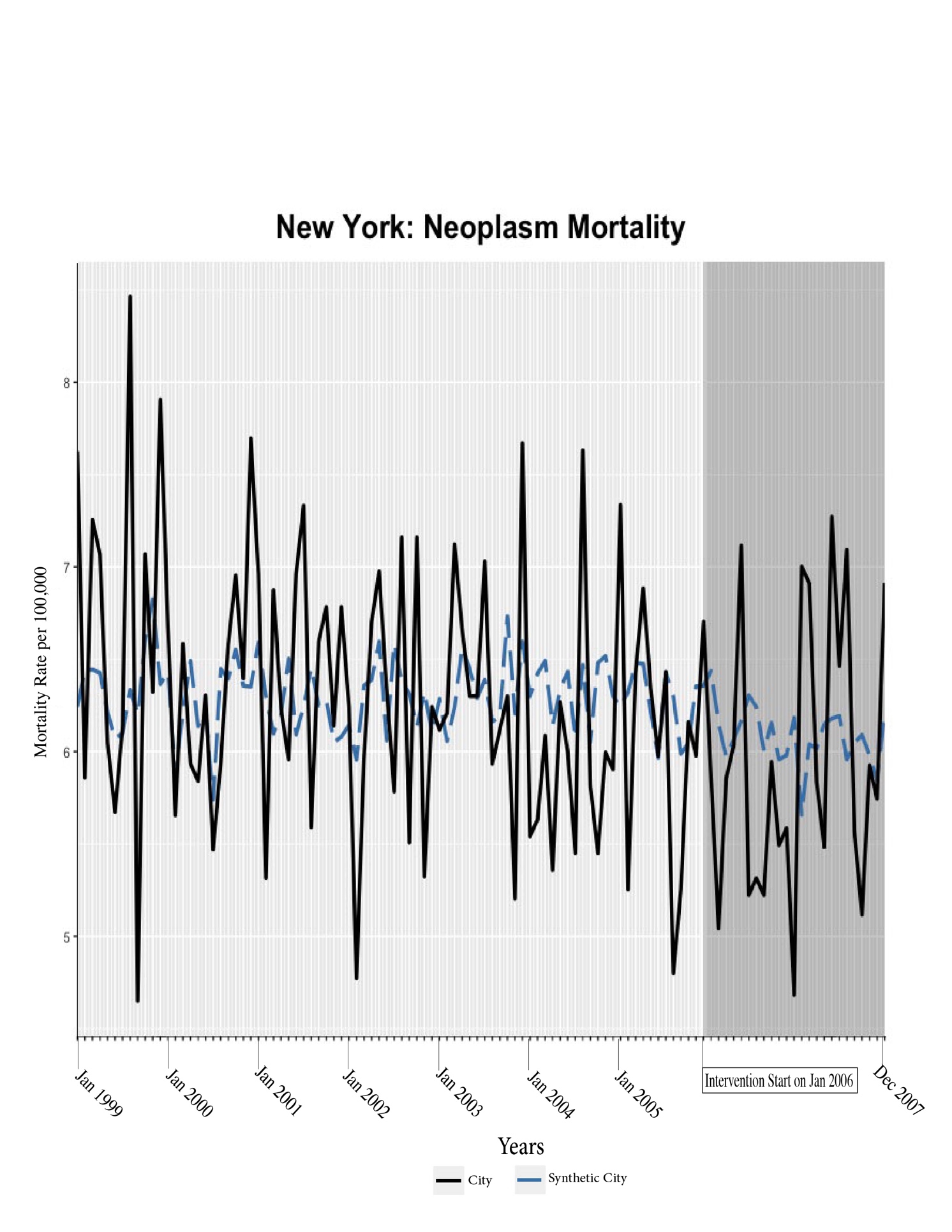

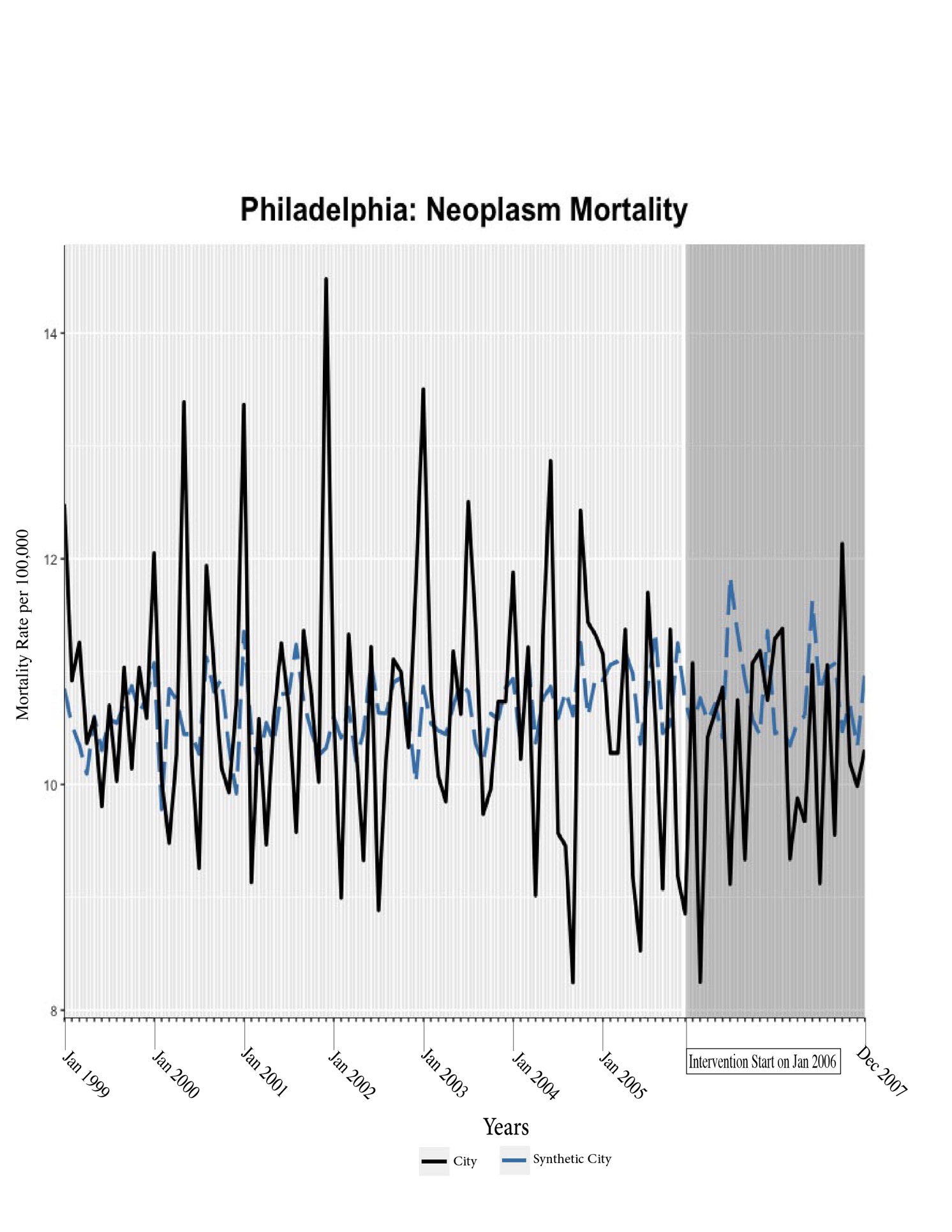

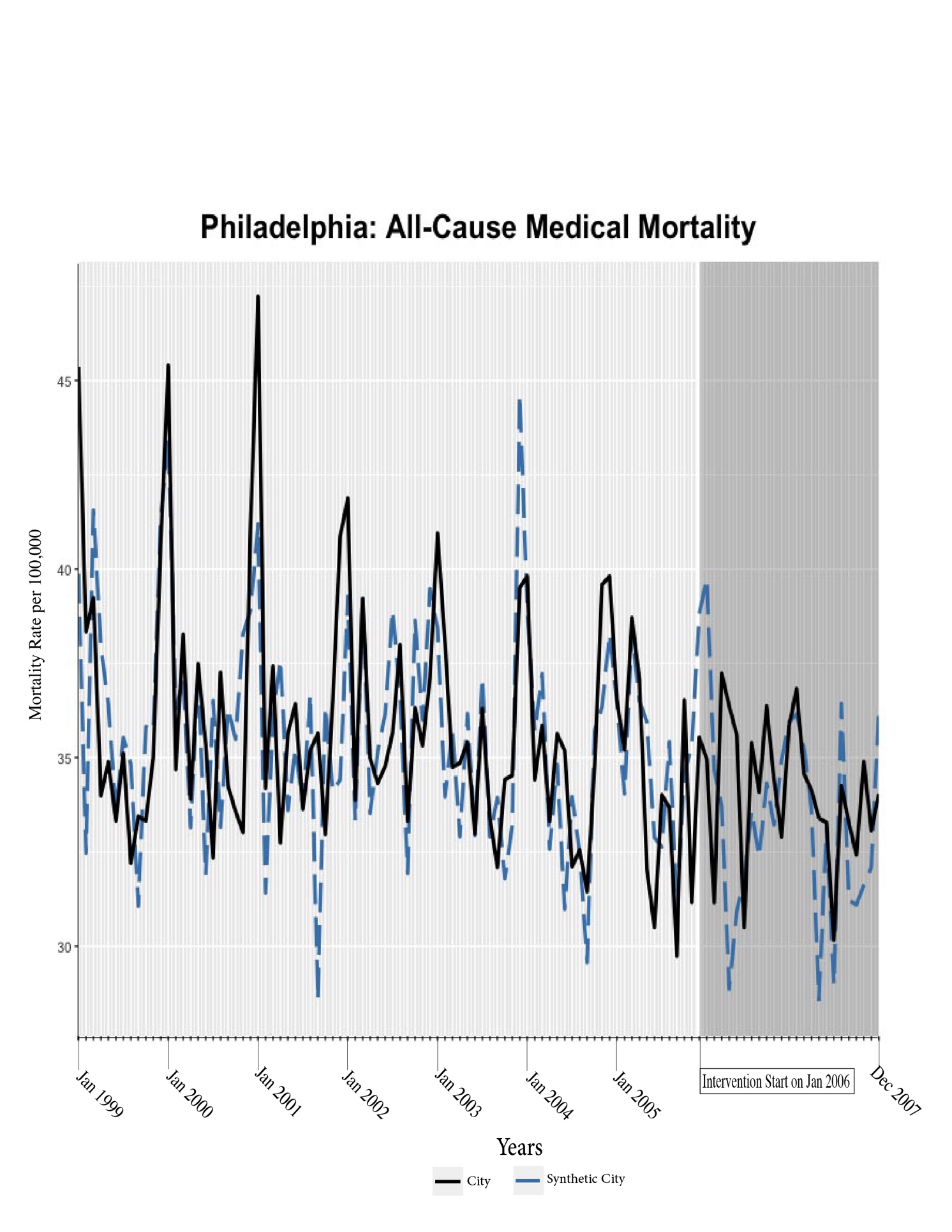

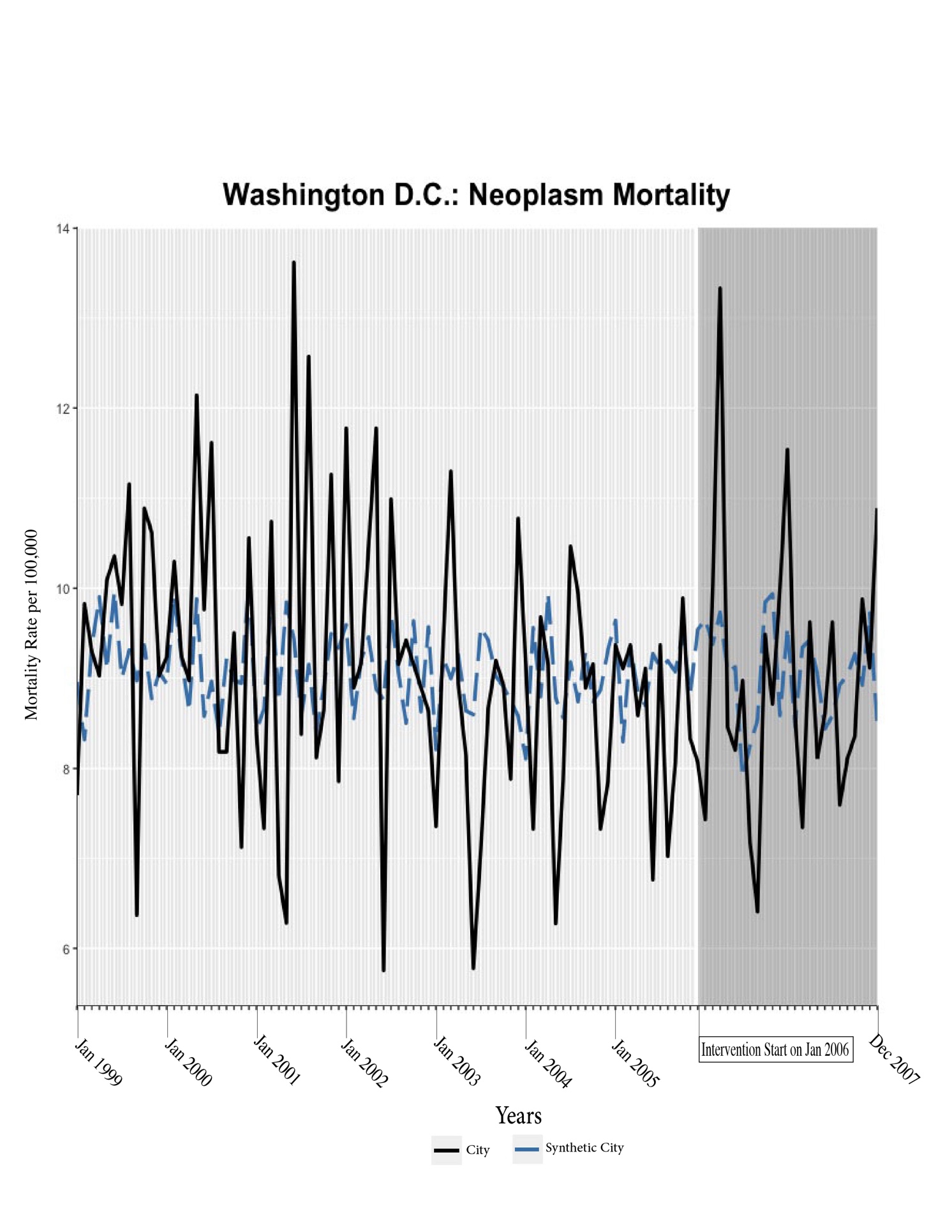


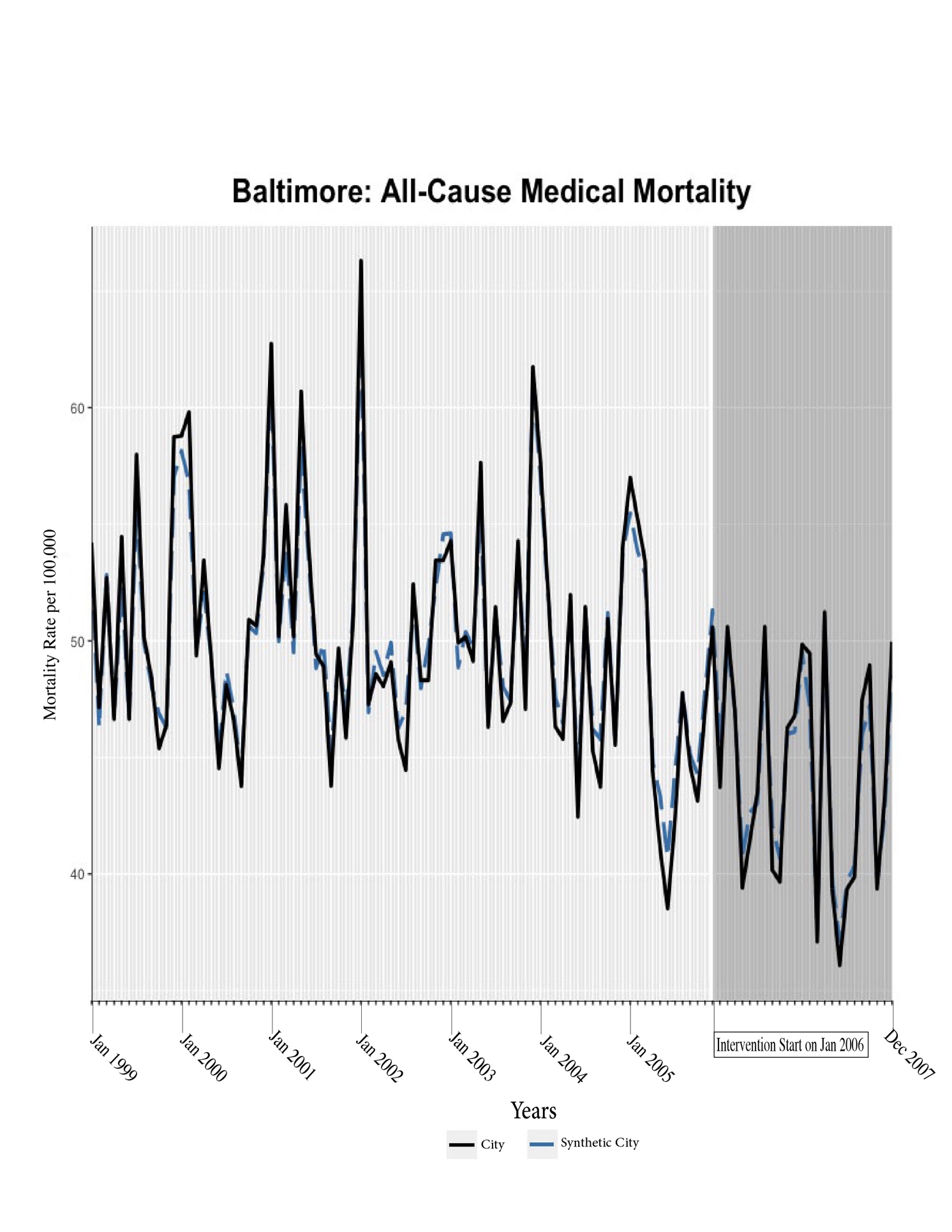

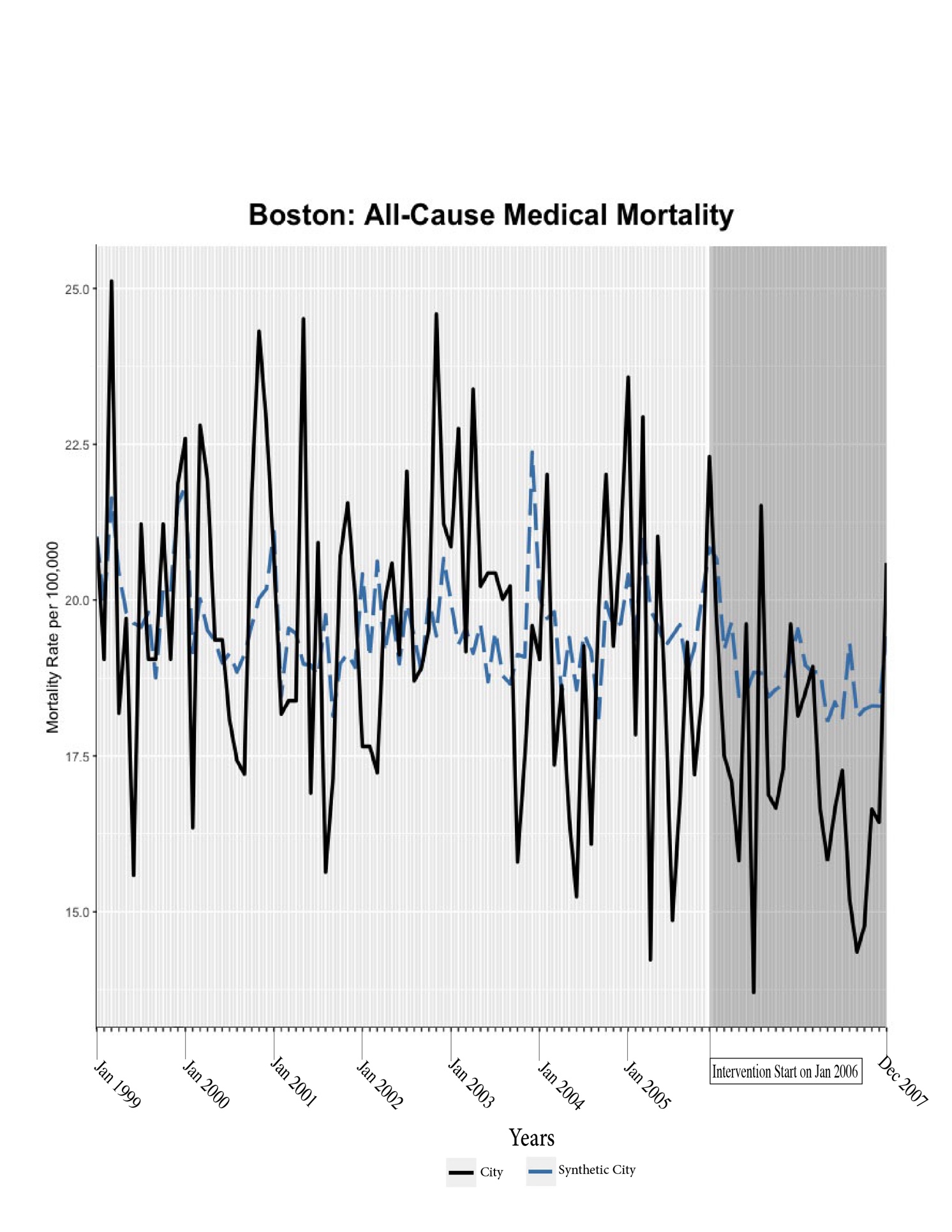

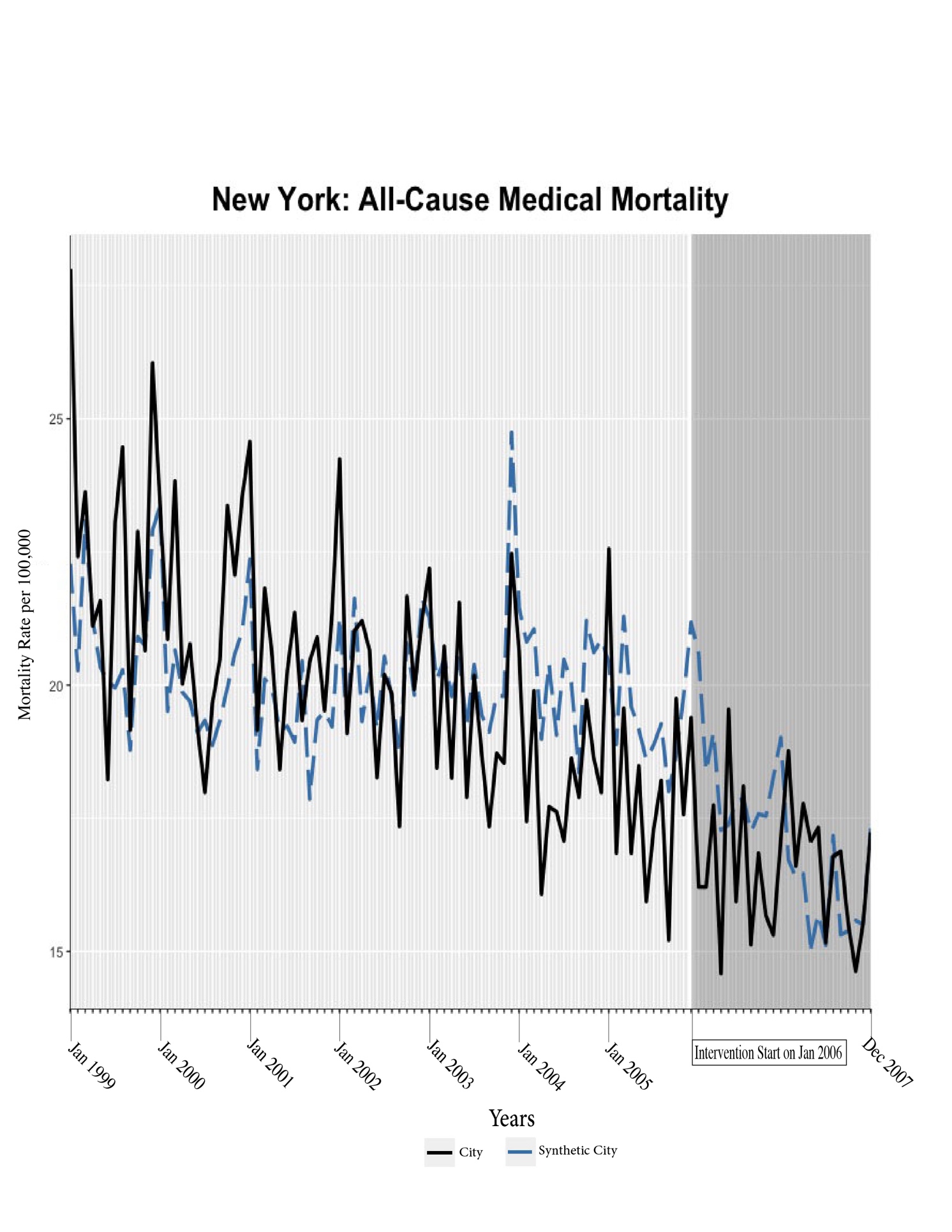

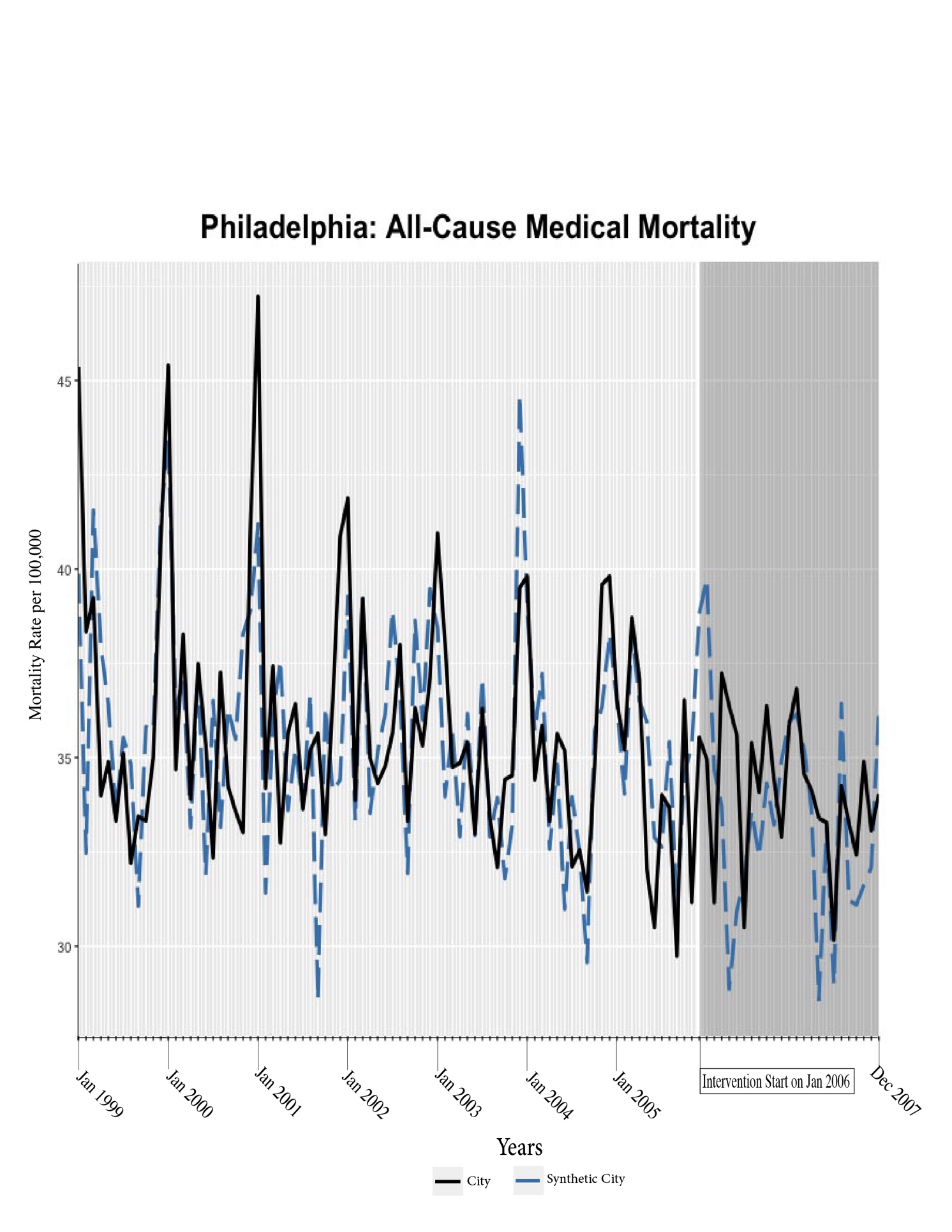

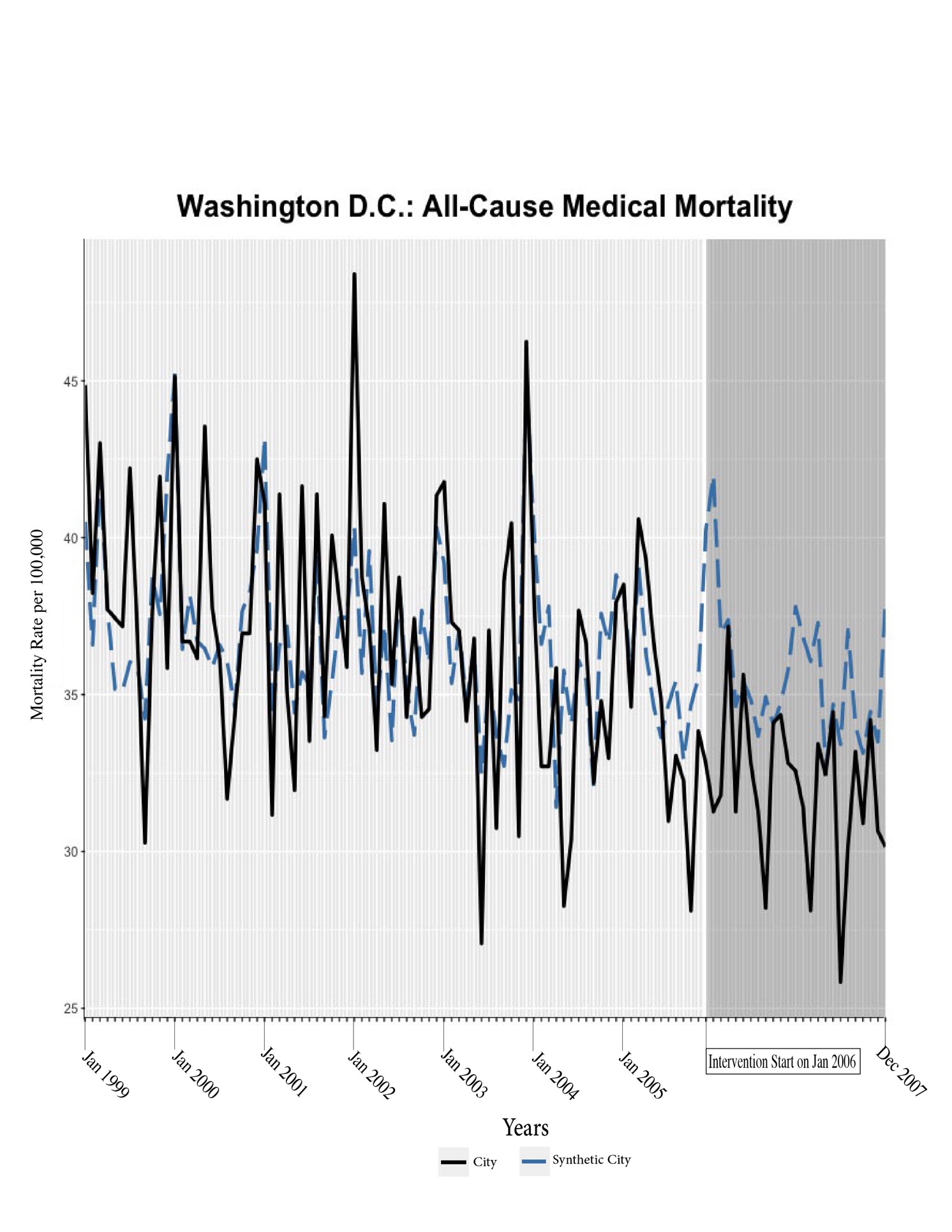


Legend: The blue dotted lines represent the generalized synthetic control prediction (synthetic city) of mortality rates while the solid black line is the observed mortality rate of the Medicaid expansion city. The vertical axis of the graphs represents mortality rate per 100,000 peoples. The horizontal axis of the graphs represents time units (i.e. the first month of the year). The darker grey graph areas correlate with the start and duration of the false Medicaid expansion in the respective city which correlate to January 2006 to December 2007.

Figure 20-25
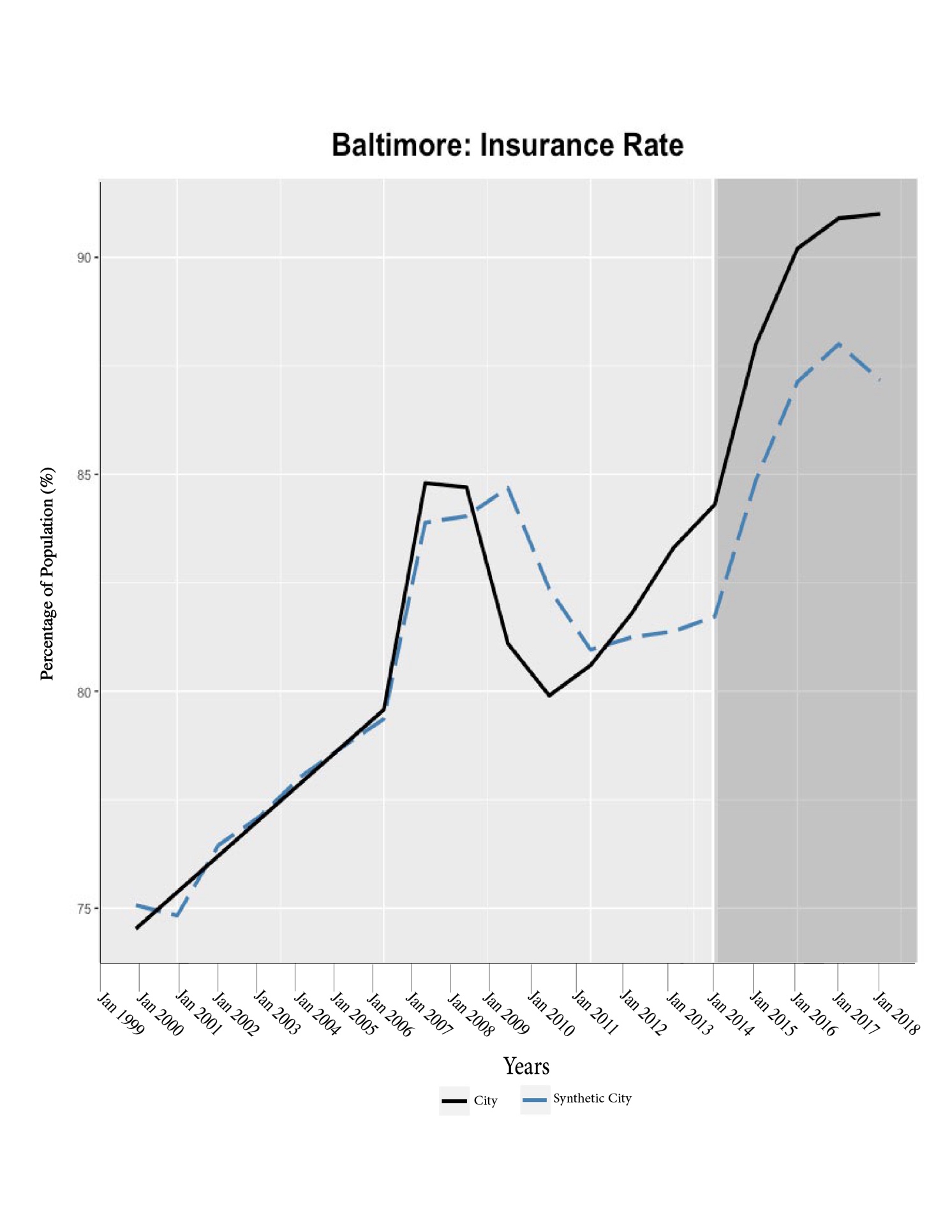

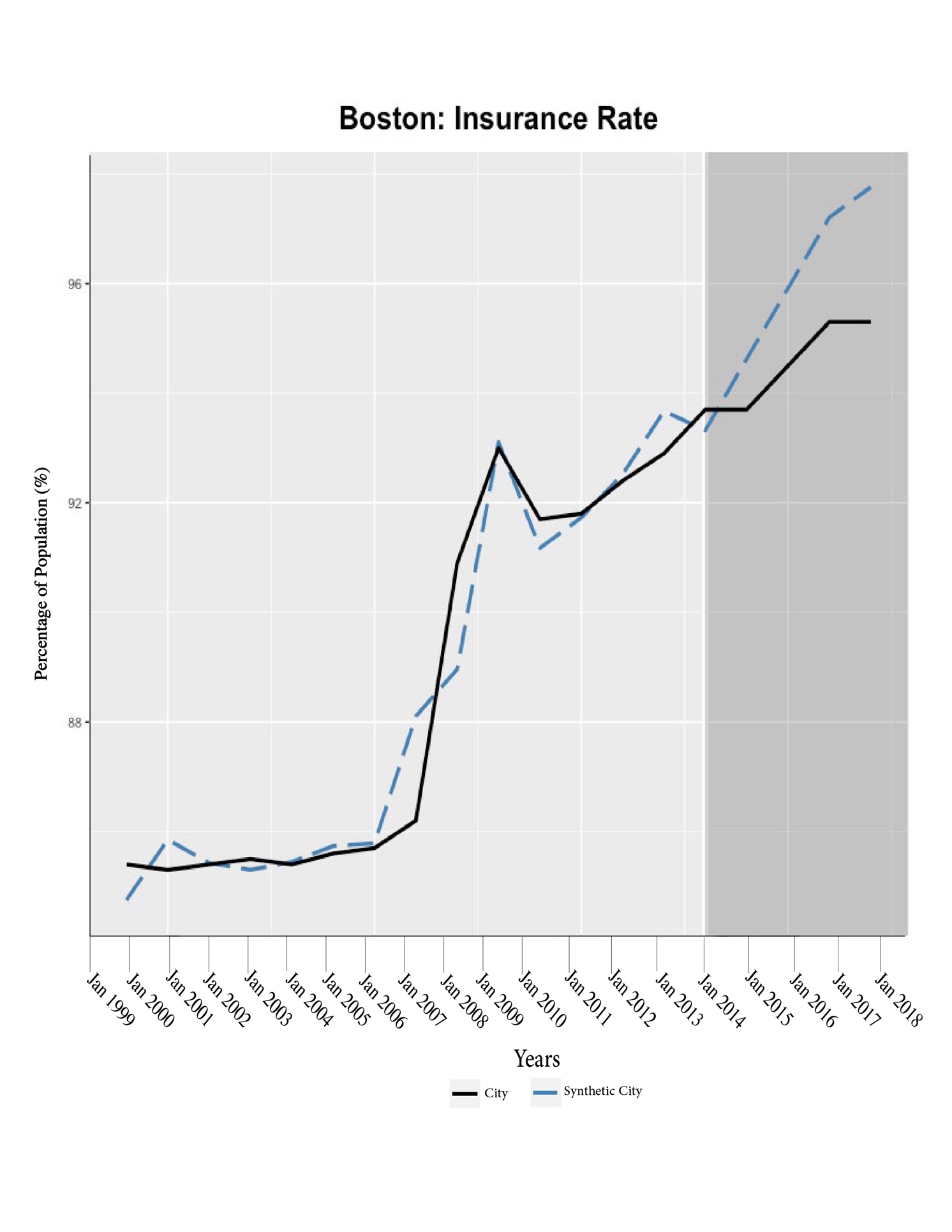

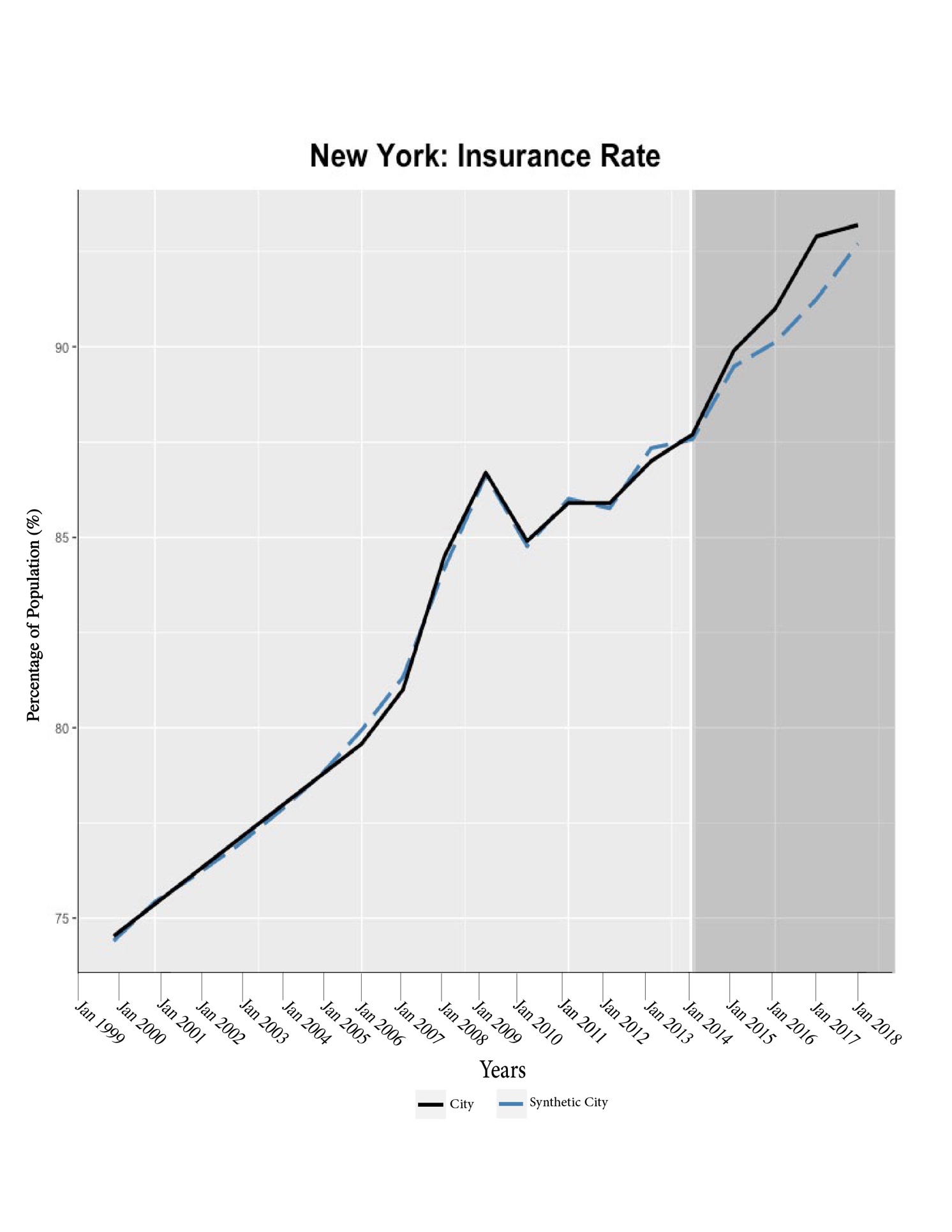

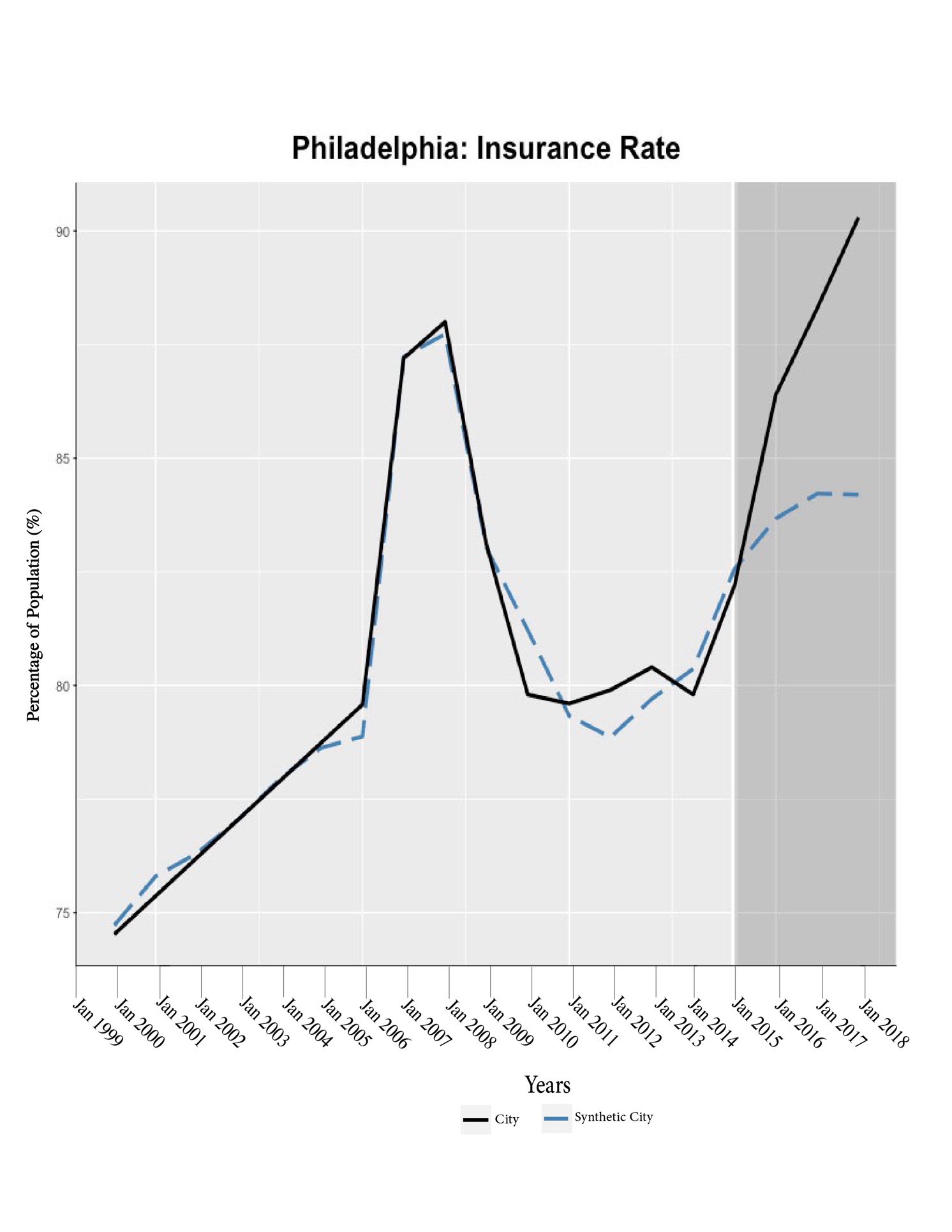

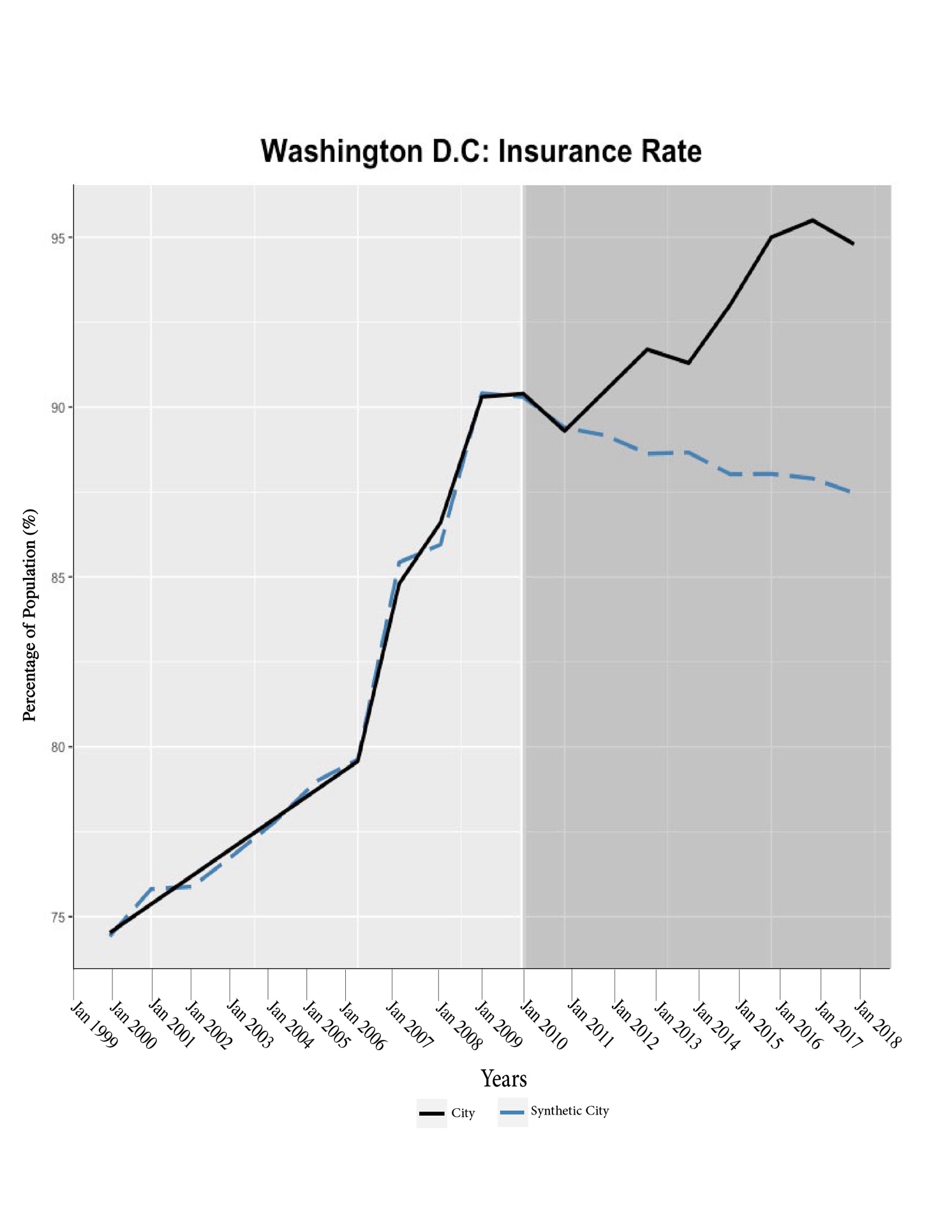


Legend: The blue dotted lines represent the generalized synthetic control prediction (synthetic city) of insurance rates while the solid black line is the observed insurance rate of the Medicaid expansion city. The vertical axis of the graphs represents the percentage insured. The horizontal axis of the graphs represents time units (i.e. the first month of the year). The darker grey graph areas correlate with the start and duration of Medicaid expansion in the respective city.
